# Asymmetric Synthesis and Biological Evaluation of Both Enantiomers of 5- and 6-Boronotryptophan as Potential Boron Delivery Agents for Boron Neutron Capture Therapy

**DOI:** 10.1021/acsmedchemlett.4c00241

**Published:** 2024-11-11

**Authors:** Michele Retini, Juulia Järvinen, Katayun Bahrami, Janne Tampio, Francesca Bartoccini, Petri Riihelä, Henna Pehkonen, Arina Värä, Tuomo Laitinen, Kristiina M. Huttunen, Jarkko Rautio, Giovanni Piersanti, Juri M. Timonen

**Affiliations:** † Department of Biomolecular Sciences, University of Urbino Carlo Bo, Piazza Rinascimento 6, 61029 Urbino, Italy; ‡ School of Pharmacy, University of Eastern Finland, P.O. Box 1627, FI-70211 Kuopio, Finland; § Applied Tumor Genomics Research Program, Faculty of Medicine, University of Helsinki, FI-00014 Helsinki, Finland; ∥ Drug Research Program, Division of Pharmaceutical Chemistry and Technology, Faculty of Pharmacy, University of Helsinki, Viikinkaari 5E, P.O. Box 56, FI-00014 Helsinki, Finland

**Keywords:** BNCT, Boron neutron capture therapy, LAT1, Large neutral amino acid transporter 1, Asymmetric synthesis, Tryptophan, BPA, *p*-Boronophenylalanine, Molecular dynamics

## Abstract

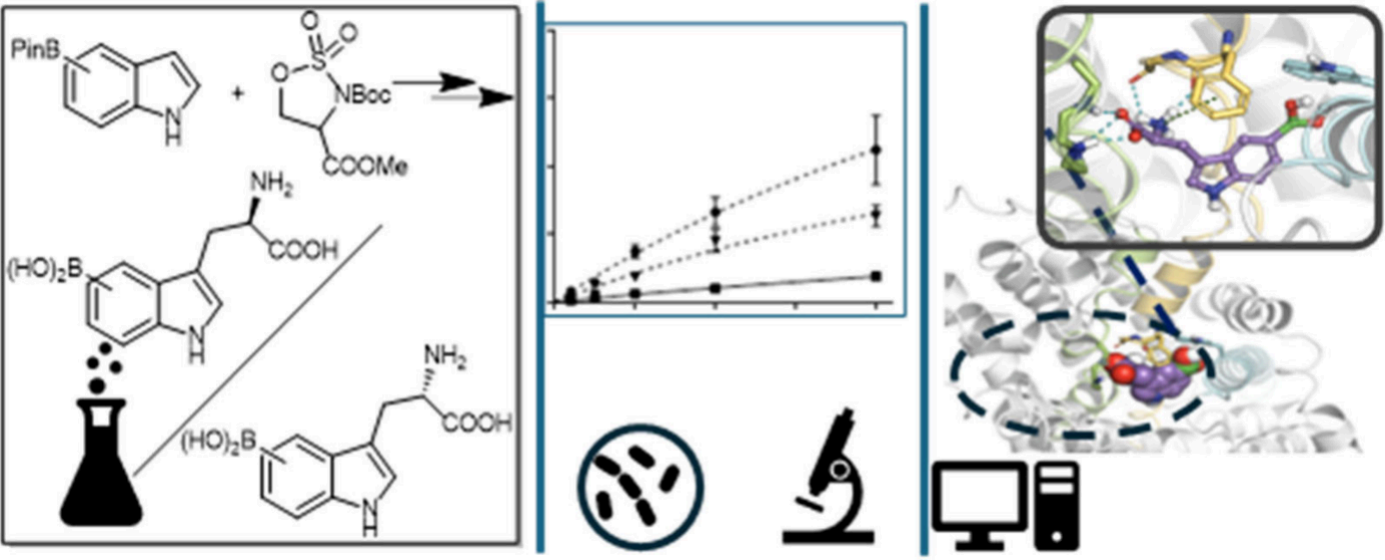

This research investigates boronated tryptophans as potential boron delivery agents for boron neutron capture therapy (BNCT) of cancer. We synthesized both enantiomers of 5- and 6-boronotryptophans (**1a** and **1b**) using simple and inexpensive methods. Their uptake was assessed in two human cancer cell lines, CAL27 (head and neck cancer) and U87-MG (brain cancer), and compared to l-*p*-boronophenylalanine (l-BPA) as a reference. To determine whether these tryptophan derivatives are substrates for large amino acid transporter 1, we performed molecular dynamics simulations to explore their transport mechanism. Our findings reveal differences in boron compound accumulation between the cancer cell lines, indicating that tryptophan derivatives could serve as effective boron carriers when the clinically used boron carrier, BPA, is ineffective.

Boron neutron capture therapy (BNCT) is an elegant radiation therapy for malignant tumors based on the ^10^B isotope’s ability to capture thermal neutrons and become radioactive.^1−3^ In BNCT, patients receive tumor-targeting boron carriers containing nonradioactive ^10^B, followed by neutron radiation. This turns ^10^B into an unstable ^11^B isotope, which decays, releasing an α particle, a Li^+^ ion, and energy. This high-energy burst can damage cell compartments and cause cell death if enough ^10^B accumulates in the cell.^1,4,5^ Despite research since the 1950s, only a few boron carriers can accumulate sufficient ^10^B in tumor cells.^6^ Clinically, only sodium borocaptate (BSH) and l-*p*-boronophenylalanine (l-BPA) are used, but they have limitations in solubility and effectiveness.^7−9^ Advances in in-hospital accelerator-based BNCT have renewed interest in developing better boron carriers.^10^ In 2020, Yu et al. reported fully protected racemic tryptophan derivatives carrying boron atoms with good tumor accumulation and selectivity but poor water solubility.^11^ However, the chemoselective asymmetric synthesis of highly water-soluble, unprotected boronotryptophans remains a daunting task.

Large amino acid transporter 1 (LAT1, *SLC7A5*) is part of a system providing essential nutrients to cells.^12,13^ LAT1 forms a complex with 4F2 heavy chain (4F2hc, *SLC3A2*),^12^ stabilizing and facilitating LAT1’s function on the plasma membrane.^14^ LAT1 transports neutral amino acids across biological barriers, e.g., the blood–brain barrier (BBB),^15^ and has been shown to be highly expressed in several cancers, including glioblastoma and head and neck cancers. This has led to the design of anticancer drugs targeting LAT1. l-BPA is somewhat transported into cancer cells via LAT1^2^ but also taken up by LAT2 (*SLC7A8*) and ATB^0,+^ (*SLC6A14*),^16^ both of which are expressed in normal tissues, especially when the concentration of l-BPA is increased.^17^ Therefore, new boron carriers with better LAT1 selectivity and effective cancer cell uptake are needed. LAT1 preferentially transports large branched and aromatic neutral amino acids, such as l-leucine and l-tryptophan, to proliferating cells.

In 2012, the Piersanti group reported a practical synthetic approach to racemic free boronic acid tryptophans using a regio- and chemoselective Lewis acid-promoted Friedel–Crafts alkylation of boronated indoles with prochiral N-protected dehydroalanine followed by mild deprotection.^18^ Unfortunately, both chiral resolution and the use of a nonracemic dehydroalanine derivative approach failed to give the desired enantiopure compounds.

Significant progress in synthesizing enantiopure free boronic acid tryptophans has been made through biocatalysis. Arnold et al.^19,20^ used directed evolution on tryptophan synthase β-subunit (TrpB) from *Pyrococcus furiosus* and *Thermotoga maritima*, creating catalysts that form C–C bonds between l-serine and various indoles, including 5- and 6-indolylboronic acids. Despite these relevant advancements, this method requires exclusive reagents, protein engineering knowledge, and specific procedures for each enantiomer.

Alternatively, enantiopure unnatural tryptophans can be accessed by alkylating indole with chiral electrophilic synthons derived from proteinogenic amino acids like serine.^21−24^ This approach allows control over the stereocenter of the electrophile, enabling access to both enantiomers without the development of new asymmetric catalytic platforms. This requires a mild, practical protocol that is tolerant of the unique electronic structure of boron. With these considerations in mind, we aimed to develop a process for the C3-alkylation of (1*H*)-indole-5- and 6-boronates (**2a** and **2b**) to obtain enantiopure l- and d-boronotryptophan derivatives utilizing the electrophilicity of chiral serine-derived cyclic sulfamidates l-**3** and d-**3** for a regioselective and stereospecific ring-opening reaction with an appropriate nucleophile.^25−33^

Since both enantiomeric forms of the amino acid serine and a variety of its protected forms are available with high optical purity at a relatively low cost, they are attractive starting materials. Consequently, cyclic sulfamidates l-**3** and d-**3** were readily synthesized from commercially available l-*N*-Boc-serine and d-*N*-Boc-serine in two steps: cyclization with SOCl_2_ and RuCl_3_-catalyzed oxidation.^31^

Conditions for the reaction of cyclic sulfamidates with heteroatom nucleophiles to produce unnatural N-protected amino acids with high optical purity have been reported.^25,32,33^ However, reactions with carbon nucleophiles have been less explored. Initial trials with hard nucleophiles resulted in complex mixtures or decomposition products. Better results were obtained with stabilized (soft) carbon nucleophiles such as β-keto esters, diethyl malonates, and phosphonate-stabilized enolates.^26−32^ With the same logic, we were able to perform the indole alkylation with **2a** and **2b** exclusively at C3 of indole with cyclic sulfamidates l-**3** and d-**3** to give orthogonally N- and C-protected boronotryptophans l-**4a**/l-**4b** and d-**4a**/d-**4b**, respectively, as single enantiomers in decent yields (Scheme 1). The key to the successful reaction outcome was softening the nucleophilic 3-position of indole prepared with MeMgCl, used as a base, in the presence of a stoichiometric amount of CuCl.

**Scheme 1:**
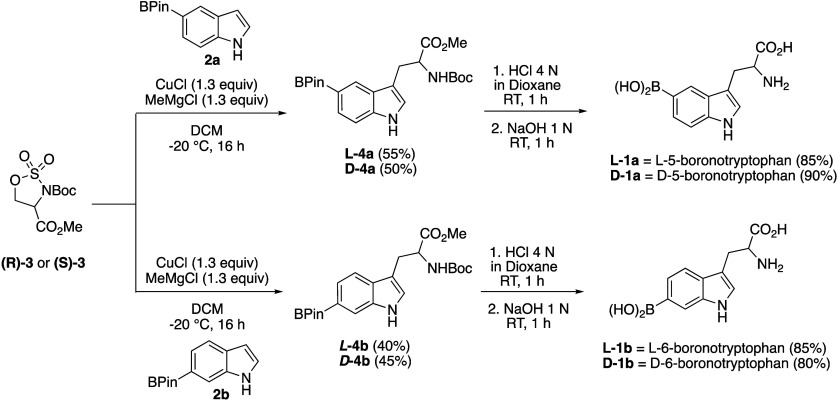
Friedel–Crafts Alkylation of Boronated Indoles **2a** and **2b** with l-**3** and d-**3** and Deprotection/Hydrolysis of Compounds l-**4a**/l-**4b** and d-**4a**/d-**4b**

To complete the synthesis of free boronotryptophans, the protecting groups were removed. The Boc protecting group was selectively removed using 4 N HCl in dioxane, avoiding protodeborylation. The resulting amine hydrochloride underwent aqueous workup and subsequent ester cleavage (both pinacolboronic and methyl carboxylic esters) with 1 N sodium hydroxide, yielding free, optically pure, amino acids l-**1a**/l-**1b** and d-**1a**/d-**1b** in high overall yields.^34^ The compounds were isolated as free bases by using normal-phase chromatography. The organic solvent was removed in vacuo, and the residual water was eliminated by lyophilization. Racemic mixtures of compounds **1a** and **1b** were synthesized using racemic sulfamidate 3 (*rac*-**3**), with spectra identical to those previously reported.^19,20^ Notably, all of the prepared boronotryptophans are soluble in water (>100 mM (24.8 mg/mL)).

For in vitro studies, we selected two human cancer cell lines, CAL27 (human oral squamous carcinoma) and U87-MG (human primary glioblastoma), both of which express LAT1.^35,36^ We screened the synthesized boronotryptophan derivatives for LAT1 affinity using a cis-inhibition assay with [^14^C]-l-leucine, similar to previous studies.^36,37^dl-BPA was used as a positive control. Despite previous findings showing increased LAT1 affinity with 5-substituted l-tryptophan,^38^ our results indicated no significant competition with [^14^C]-l-leucine for LAT1 uptake by either racemates, enantiopure derivatives, or dl-BPA (data not shown). Thus, the affinity results indicate that the compounds may lack selectivity as LAT1 substrates or weak binding potency relative to a radiolabeled substrate, [^14^C]-l-leucine, given their limited inhibitory effect on the substrate even at high concentrations. It is important to highlight that inhibitory efficiency specifically measures the compounds’ ability to bind to LAT1, without providing insight into their capacity to translocate across the cell membrane via LAT1 or other transport mechanisms. Therefore, further investigation into their uptake was conducted.

To evaluate the potential of 5-boronotryptophan (**1a**) and 6-boronotryptophan (**1b**) as boron carriers in BNCT, we studied their cellular uptake in CAL27 and U87-MG cells at concentrations from 5 to 200 μM. These concentrations were selected considering the proposed dose-dependent shift from LAT1 transport to other transporters at higher concentrations. To determine the optimal incubation time for concentration-dependent uptake studies, the cellular uptake of the compounds was measured at 100 μM over different time points (2, 5, 10, 15, 20, 30, 40, and 60 min). The optimal incubation time was chosen from the linear range of the uptake curve, with a 5 min incubation time selected for further studies, consistent with our previous research on boronated compounds.^39,40^

The results showed dose-dependent uptake of boronated tryptophan derivatives and dl-BPA in both cell lines, along with a notable disparity between the two cell lines. In CAL27 cells, uptake of both racemic tryptophan derivatives saturated at low concentrations, while dl-BPA did not show clear saturation and was transported more efficiently across the concentration range (Figure 1A). In U87-MG cells, both racemic tryptophan derivatives exhibited higher transport compared to dl-BPA, with no clear saturation (Figure 1B). The 5-isomer showed a greater transport efficiency in U87-MG, while the 6-isomer was more effective in CAL27 to some extent. The 5-isomer in U87 cells demonstrated 4 times greater transport efficiency than dl-BPA, whereas the 6-isomer had comparable or slightly better uptake than dl-BPA. The concentrations of the test compounds taken up by cells after incubation with varying concentrations of these compounds are shown in Table S1 along with Michaelis–Menten kinetic parameters.

**Figure 1:**
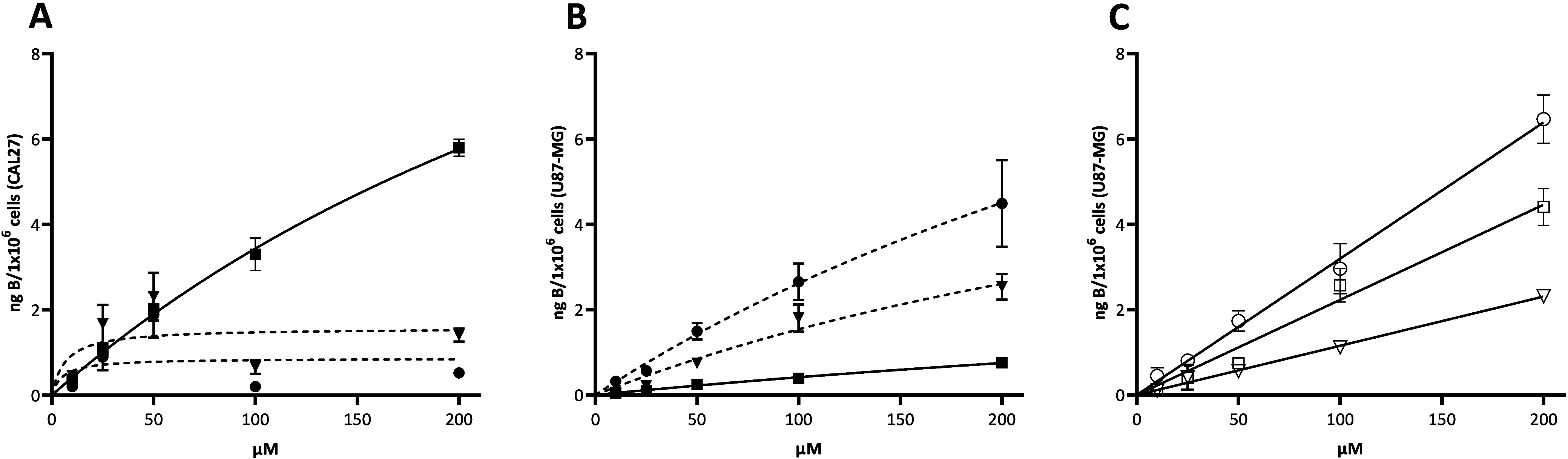
Uptake profiles of dl-5-boronotryptophan (●, dashed line), dl-6-boronotryptophan (▼, dashed line), and dl-BPA (■, solid line) in (A) CAL27 cells and (B) U87-MG cells. (C) Uptake of l-5-boronotryptophan (○, solid line), d-5-boronotryptophane (▽), and l-BPA (□) in U87-MG cells (mean ± SD, *n* = 3).

Building on these somewhat promising findings, U87-MG was chosen as a focal cell line and 5-boronotryptophan as the prime compound for further exploration. To gain a deeper understanding of the transport of these compounds and evaluate the potential of boronated tryptophan derivatives as boron carriers in BNCT, we studied the uptake of the enantiopure compounds l-5-boronotryptophan (l-**1a**) and d-5-boronotryptophan (d-**1a**), with l-BPA used as a reference. The results surpassed our expectations, with l-**1a** exhibiting higher uptake than l-BPA (Figure 1C). Notably, the l isomer outperformed its counterpart in transport, aligning with our anticipated outcomes. Additionally, l-**1a** was also shown to be nontoxic at the used doses (Figure S4). The uptake rate for the l isomer was approximately twice as high across the studied concentration range compared to l-BPA. However, differences between the results obtained from CAL27 and U87-MG led us to characterize the CAL27 cell line. Characterization of U87-MG had already been done in our previous study.^36^

## In Vitro Characterization of Cell Lines

To evaluate LAT1 function in CAL27 cells, we measured [^14^C]-l-leucine uptake under varying conditions (Figure S2). Surprisingly, [^14^C]-l-leucine uptake decreased significantly in the absence of sodium, despite LAT1 being sodium-independent. Additionally, the specific LAT1 inhibitor was ineffective, suggesting that l-leucine may use alternative transport mechanisms such as LAT2 (*SLC7A8*) or B0AT2 (*SLC6A15*) in CAL27 cells. In contrast, in U87-MG cells, the absence of sodium did not affect l-leucine uptake, and the LAT1 inhibitor was effective.^36^

Following these intriguing results, we investigated the presence and subcellular localization of the LAT1–4F2hc complex in CAL27 cells. We used immunofluorescence staining and fluorescence microscopy (Axio Imager with ApoTome.2, Carl Zeiss) to visualize hLAT1 and 4F2hc expression levels (Figure S3). LAT1 is primarily localized to the cell membrane, consistent with its role as a transporter. Previous studies have shown that 4F2hc is essential for the cellular localization, stability, and transport activity of LAT1.^12,41,42^ Our results align with these findings, showing that the localization of 4F2hc correlates with that of LAT1, although 4F2hc can also be detected within intracellular compartments, coexpressed with other transporters from the SLC family. We also quantified transporter protein levels using LC-MS/MS-SRM, measuring LAT1 at 0.31 ± 0.04 fmol/μg of protein (normalized to the membrane marker NA^+^/K^+^-ATPase) and 4F2hc at 0.16 ± 0.001 fmol/μg of protein. In U87-MG, LAT1 was 0.36 ± 0.16 fmol/μg of protein, and 4F2hc was 0.082 ± 0.014 fmol/μg of protein.^36^ This confirmed that CAL27 cells express LAT1 as expected, but l-leucine and our boronated tryptophans likely use an alternative transport route in these cells.

In the literature, l-BPA was the first boron delivery agent shown to enter cancer cells via LAT1.^2^ However, l-BPA is also transported by LAT2 and ATB^0,+^, which are expressed in normal tissues and some cancer cells.^16^ The uptake of l-BPA into cancer cells is mainly due to high-affinity transport by LAT1, but in cancer cells with high ATB^0,+^ expression, the lower-affinity uptake by ATB^0,+^ becomes significant at higher l-BPA concentrations. For CAL27, a similar alternative transport mechanism might dominate at higher l-BPA concentrations, whereas boronated tryptophans are not able to utilize these mechanisms (Figure S1).

Because the characterization of cell lines proposed different transport mechanisms in CAL27 and U87-MG and the results of the uptake study supported this, we further investigated the interactions of boronated tryptophan derivatives and l-BPA with LAT1 using molecular modeling. While compound accumulation involves several biochemical processes (e.g., uptake, efflux, and metabolism), molecular modeling offers valuable insights into whether a compound can be recognized by the transporter and the nature of ligand–protein interactions. However, it may not capture the full complexity of these processes.

## Molecular Modeling Studies

Conducting unconstrained induced-fit docking, the cryo-EM structure of LAT1 in the outward open conformation (PDB ID 7DSQ) was selected as a template.^42,43^ Selected favorable docking poses were subjected to replicated molecular dynamics simulations with membrane and solvent/ions for 5 μs.

**Figure 2:**
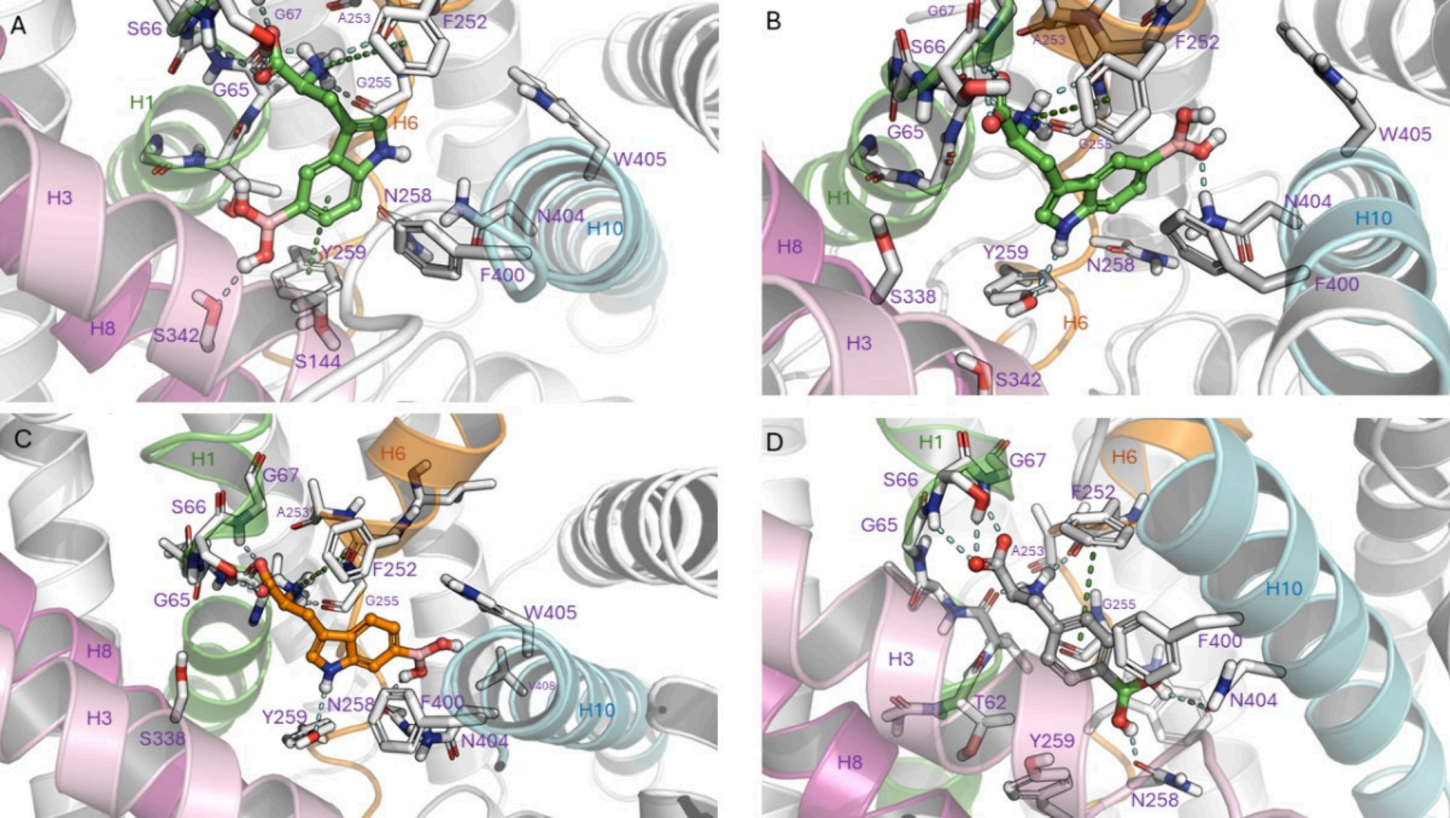
Selected representative snapshots from molecular dynamics simulations illustrating commonly detected interactions (Figures S7 and S8) of key compounds with LAT1 (PDB ID 7DSQ) close to the 2 μs time point of the replicated trajectory: (A) l-5-boronotryptophan (l-**1a**) in the proximal pocket; (B) l-**1a** in the distal pocket; (C) l-6-boronotryptophan (l-**1b**) in the distal pocket; (D) l-BPA in the distal pocket. Color depictions of transmembrane (TM) helixes: TM-1, green; TM-6, orange; TM-3, pink; TM-8, violet; TM-10, blue.

As depicted by Figures S5 and S6, all the simulated systems were stable. Focusing on the interactions throughout the simulation, we noticed that similar to another docking study of l-BPA with the homology model of LAT1,^44^ the amino acid moiety of all ligands was grabbed by the conserved residues of the recognition site (TM1 and TM6) of the transporter. In our study, we observed that the rest of the ligands’ structure was accommodated in the distal cavity between TM6 and the upper part of TM10 and TM3 with the boronic acid moiety moving and establishing intermittent hydrogen bonds with N404 and N258 of TM10 and TM6 (Figures 2 and S9). In addition to the π–π interaction between F400 of TM10 and the aromatic ring of all compounds, we observed further hydrogen-bond or π–π interactions of the indole ring of both l-5-boronotryptophan and l-6-boronotryptophan with Y259 in the deeper part of TM6 of the transporter (Figure 2A–C). To obtain a wider perspective, we conducted one more simulation for l-5-boronotryptophan with its indole ring placed in the proximal cavity surrounded by TM1 and the upper part of TM8 and TM3 and observed the boronic acid moiety making alternate hydrogen bonds with S338, S342, and S144 of TM8 and TM3 (Figure 2A). The most common protein–ligand interactions throughout the simulation are depicted in Figures S7 and S8.

**Figure 3:**
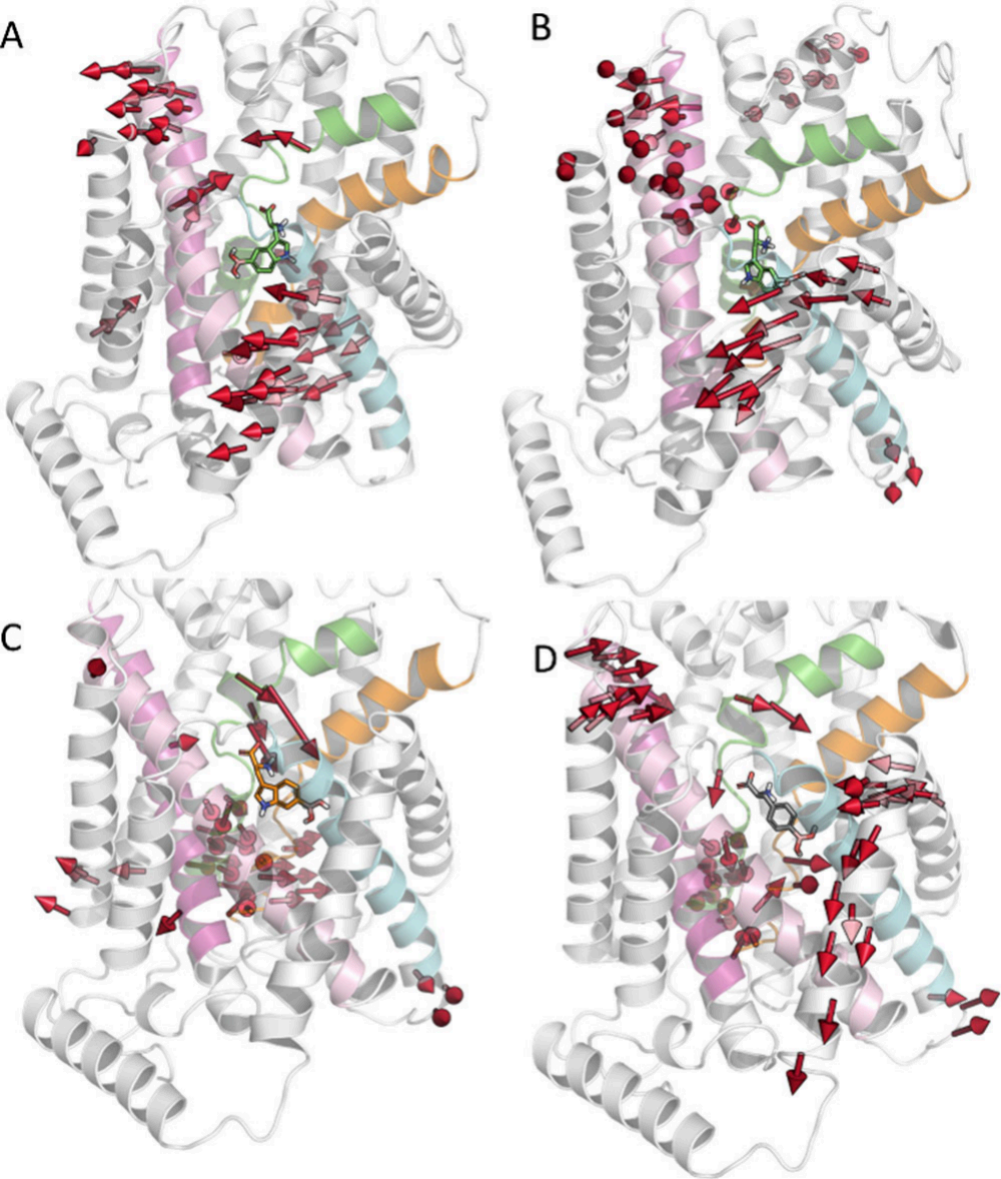
Essential motions of TM helices according to PC1 for (A) l-5-boronotryptophan in the proximal pocket, (B) l-5-boronotryptophan in the distal pocket, (C) l-6-boronotryptophan, and (D) l-BPA. Specific color depictions for transmembrane helixes: TM-1, green; TM6, orange; TM-3, pink; TM-8, violet; and TM-10, blue.

Next, to identify the essential motions of the transmembrane domains, principal component analysis (PCA) was performed considering the residues inside helices (Figure S10). Our observations revealed that all three compounds disrupted the secondary structure of TM6b, which is an important feature associated with the inward-open conformation. The most extensive motion with l-BPA occurred in the hash domain (TM3 and TM8), TM12, and the bundle domain (TM1a and TM6b) (Figure 3D). l-5-Boronotryptophan accommodated either in the proximal or distal cavity induced similar movement as l-BPA in TM3, TM8, and TM12. In addition to the motions of TM6b, the compound also disrupted the secondary structure of TM2 and TM7 of the bundle domain when located in the proximal cavity (Figure 3A,B). The motions of the latter two helices, which are in close contact with the main transportation path (TM1 and TM6), prepare the protein for adopting the inward-open state. Interestingly, the most significant motions with l-6-boronotryptophan were displayed only in the TM1a and TM6b of the bundle domain (Figure 3C). The motion of TM12 that was observed with both l-BPA and l-5-boronotryptophan is understood to be involved in accommodating a cholesterol molecule that is required for the activity of LAT1.^45^

In light of findings from uptake studies in U87-MG cells, it can be concluded that enhanced interactions of tryptophan derivatives observed in MD simulations might have contributed to their higher uptake relative to l-BPA. However, we witnessed that l-6-boronotryptophan, despite maintaining stable interactions with deeper parts of LAT1 and affecting the conformation of the main transportation path in PCA analysis, exhibited a lower uptake than l-5-boronotryptophan in U87-MG cells. One explanation may lie in the dynamic nature of the transportation process, which requires the concomitant movement of different parts of the protein. In this respect, a loosely bound ligand like l-5-boronotryptophan, by inducing coupled motions in the hash and bundle domain, might trigger a smoother helix translocation that could finally lead to the ligand release, whereas maintaining more stable interactions and inducing motions in the bundle domain only, as observed with l-6-boronotryptophan, might have contributed to a more confined pose that slows down the helix translocation process. Taking the idea of the loosely bound pose into account, it is also probable that the small size and smaller interactions of l-BPA lead to its transport not only by LAT1 but also by other transporters. Its significantly higher uptake compared to tryptophan derivatives in CAL27, regarding the low functionality of LAT1 in these cells, implies its lower selectivity for LAT1.

## Conclusion

To be effectively utilized, BNCT requires novel boron carriers that meet commonly accepted criteria: (1) sufficient accumulation of the boron-10 isotope in tumor tissue, (2) a favorable blood/tumor and healthy tissue/tumor ratio, and (3) suitable pharmacokinetic properties. Currently, clinically used boron carriers do not fulfill these criteria. In this study, we aimed to develop novel boronated tryptophan derivatives capable of efficiently delivering boron into tumor cells.

A straightforward procedure for the chemical synthesis of both (d and l) enantiomerically pure unprotected 5- and 6-boronotryptophans from simple indole starting materials and a suitable and readily available chiral serine-derived sulfamidate was developed. In addition, it was shown that these compounds are transported into two tumor cell lines, U87-MG and CAL27, known to express several amino acid transporters, including LAT1. Notably, these studies indicated that CAL27 cells exhibit the non-LAT1 transport of l-leucine. Given the knowledge that tryptophan and its diverse non-boronated derivatives serve as LAT1 substrates, the current study also illustrates, through in silico analysis, the potential of the novel boronated compounds to function as LAT1 substrates. It was noted that all three compounds, l-BPA, l-5-boronotryptophan, and l-6-boronotryptophan, were recognized by LAT1 and induced the inward-open conformation. However, by establishing intermittent interactions with residues of TM6 and TM10, tryptophan derivatives may lead to more LAT1-specific uptake. These modeling studies also suggest that the superiority of l-5-boronotryptophan over l-6-boronotryptophan in uptake studies may arise from its ability to spur motions in different helices, most probably via the generation of a loosely bound conformation. However, it is worth pondering that the results of MD simulations are dramatically affected by the starting pose and may not solely reflect the whole transportation process.

Collectively, these results propose that l-5-boronotryptophan is a potent boron carrier in tumors, where l-BPA is not efficiently transported. The results can be very beneficial, especially when interindividual genetic variations in transporter expression are considered in selecting the drug of choice in personalized therapy in the future. Therefore, this study introduces a new landscape for the development of boron carriers for BNCT and adds these tryptophan derivatives and synthesis methods to the toolbox of medicinal chemists working in the field of boron carrier development.

## Supporting Information Available

Synthesis and characterization of compounds, experimental methods, and CAL27 cell line characterization by LAT1 function; uptake profiles with Eadie–Hofstee plots (Figure S1); CAL27 LAT1 function (Figure S2); fluorescence microscopy and transporter protein expression in CAL27 cells (Figure S3); impact of l-**1a** on Caco-2 cell viability (Figure S4); MD simulation results (Figures S5–S10) (PDF)

## Abbreviations

4F2hc: 4F cell surface antigen
ATB^0,+^: amino acid transporter B^0,+^
BBB: blood–brain barrier
BNCT: boron neutron capture therapy
BOC: *tert*-butoxycarbonyl
BPA: 4-boronophenylalanine
BSH: sodium mercaptoundecahydro-*closo*-dodecaborate
CAL27: oral adenosquamos carcinoma cell line
cryo-EM: cryogenic electron microscopy
DCM: dichloromethane
EtOAc: ethyl acetate
HPLC: high-performance liquid chromatography
HRMS: high-resolution mass spectrometry
LAT1: large amino acid transporter 1
LC-MS/MS-SRM: liquid chromatography–tandem mass spectrometry selected reaction monitoring
MD: molecular dynamics
NMR: nuclear magnetic resonance
PDB: Protein Data Bank
ppm: parts per million
*rac*: racemic
Q-TOF: quadrupole time-of-flight
SLC: solute carrier
THF: tetrahydrofuran
TLC: thin-layer chromatography
TrpB: tryptophan synthase β-subunit
U87-MG: glioma cell line
