# Supplementary material for: Asymmetric Synthesis and Biological Evaluation of Both Enantiomers of 5- and 6-Boronotryptophan as Potential Boron Delivery Agents for Boron Neutron Capture Therapy
Source: ACS Med Chem Lett. 2024 Nov 11;15(12):2121–8. doi: 10.1021/acsmedchemlett.4c00241 (PMC11647679; doi:10.1021/acsmedchemlett.4c00241)
Supplement: Supplementary file 1 — ml4c00241_si_001.pdf [file ml4c00241_si_001.pdf]

SUPPORTING INFORMATION

Asymmetric synthesis and biological evaluation of both enantiomers of 5- and 6-boronotryptophan as potential boron delivery agents for boron neutron capture therapy (BNCT)

Michele Retini[1], Juulia Järvinen[2], Katayun Bahrami[2], Janne Tampio[2], Francesca Bartoccini[1], Petri Riihelä[2], Henna Pehkonen[3], Arina Värä[3], Tuomo Laitinen[2], Kristiina M. Huttunen[2], Jarkko Rautio[2], Giovanni Piersanti[1], Juri M. Timonen[2,4*]

[1] Department of Biomolecular Sciences, University of Urbino Carlo Bo, Piazza Rinascimento 6, 61029 Urbino, PU, Italy

[2] School of Pharmacy, University of Eastern Finland, P.O. Box 1627, FI-70211 Kuopio, Finland.

[3]Applied Tumor Genomics Research Program, Faculty of Medicine, University of Helsinki, Finland,

[4] Drug Research Program, Division of Pharmaceutical Chemistry and Technology, Faculty of Pharmacy, University of Helsinki, Viikinkaari 5E, P.O. Box 56, 00014 Helsinki, Finland. juri.timonen@helsinki.fi.

Contents

1. Supporting methods 2
   1.1. Synthesis 2
   1.2. Biological evaluation 16
   1.3. Analytical methods 19
   1.4. Molecular modeling 19
2. Characterization of CAL27 line 20
   2.1. LAT1 function 20
   2.2. Fluorescence microscopy imaging 20
   2.3. Western blot 21
3. Cytotoxicity study of the key compounds 23
4. Supporting Figures from Molecular Dynamics Simulations 24
5. References 27

1. Supporting methods

1.1. Synthesis

General Information All reagents were purchased from commercial suppliers and used without further purification. Column chromatography purifications were performed in flash chromatography conditions using 230-400 mesh silica gel and 70-230 mesh silica gel for free 5- and 6- tryptophan boronic amino acids. Analytical thin layer chromatography (TLC) was carried out on silica gel plates (Silica Gel 60 F254). ^{1}H NMR, ^{13}C NMR, and ^{11}B NMR spectra were recorded on 400 MHz spectrometer using $CDCl_3$ and D_2O as solvents. Chemical shifts (δ scale) are reported in parts per million (ppm) relative to the central peak of the solvent. Coupling constants (J values) are given in hertz (Hz). In ^{13}C NMR carbon adjacent to boron was not observed.[1–3] All the final compounds tested (**L-1a**, **D-1a**, **L-1b**, **D-1b**) had purity greater than 95%, judged by ^{1}H NMR spectroscopy and high-pressure liquid chromatography. HPLC analyses were performed on a Waters HPLC/UV/MS system (separation module Alliance HT2795, photo diode array detector 2996, mass detector Micromass ZQ, using column Phenomenex C6-phenyl 150 mm × 4.60 mm × 5 mm. The mobile phase consisted of acetonitrile and water (e containing 0.1% formic acid). A linear gradient of 70% to 100% acetonitrile over 8 minutes was used with a 10-minute run time at a flow rate of 1 mL/min. Optical rotation analysis was performed with a polarimeter using a sodium lamp (λ = 589 nm, D line), $[\alpha]_D^{25}$ values are reported in 10^{-1} deg cm^2 g^{-1}, and concentration (c) is in g/100 mL. HRMS analysis was performed using a Q-TOF microTM mass spectrometer.

Starting Material. Indole derivatives (**2a-b**) are commercially available. 3-(tert-butyl) 4-methyl (R)-1,2,3-oxathiazolidine-3,4-dicarboxylate 2,2-dioxide (**(R)-3**) and 3-(tert-butyl) 4-methyl (S)-1,2,3- oxathiazolidine-3,4-dicarboxylate 2,2-dioxide (**(S)-3**) were synthetized as reported in literature.[4] Optical rotation analysis: (**(R)-3**) $[\alpha]_D^{25}$ = +27 (c = 1.03, $CHCl_3$), (**(S)-3**) $[\alpha]_D^{25}$ = -26.7 (c = 0.99, $CHCl_3$).

General procedure for indole C3-alkylation. To a suspension of the appropriate indole derivatives (**2a,b**) (1.5 equiv) and CuCl (1.3 equiv) in DCM dry (0.3 M) at 0 °C was added dropwise MeMgCl (3 M in THF, 1.3 equiv) over 10 min. The reaction mixture was stirred at 0 °C for 1 h and cooled to -20 °C. A solution of **L-3** or **D-3** (1 equiv) in DCM (0.85 M) was added into the reaction mixture over 20 min dropwise at -20 °C. The reaction was allowed to return to room temperature and stirred for 16 h. The reaction was diluted with DCM (10 mL for 1.0 mmol of **L-3** or **D-3**) and 2 M citric acid solution (10 mL for 1.0 mmol of **L-3** or **D-3**) was added dropwise at 0 °C and then was stirred at room temperature for 10 min, filtered on Celite, and the phases were separated. The aqueous phase was extracted with DCM (3 x 10 mL for 1.0 mmol), washed with brine (30 mL for 1.0 mmol), dried over Na_2SO_4, filtered, and purified by flash chromatography.

Methyl (S)-2-(tert-butoxycarbonyl)amino)-3-(5-(4,4,5,5-tetramethyl-1,3,2-dioxaborolan-2-yl)-1H-indol-3-yl)propanoate (L-4a) The general procedure was performed between 5-(4,4,5,5-tetramethyl-1,3,2-dioxaborolan-2-yl)-1*H*-indole (**2a**) (364.6 mg, 1.5 mmol) and **L-3** (281 mg, 1 mmol). The crude product was purified by flash chromatography (cyclohexane/EtOAc = 8:2) to yield **L-4a** (222 mg, 50%) as a white solid. ^{1}H NMR (400 MHz, $CDCl_3$) δ 8.30 (br s, 1H), 8.06 (d, *J* = 1.0 Hz, 1H), 7.64 (dd, *J* = 8.0, 1.0 Hz, 1H), 7.33 (d, *J* = 8.0 Hz, 1H), 6.99 (s, 1H), 5.10 (br d, *J* = 8.0 Hz, 1H), 4.69–4.64 (m,1H), 3.73 (s, 3H), 3.32 (m, 2H), 1.43 (s, 9H), 1.37 (s, 6H), 1.36 (s, 6H). ^{13}C NMR (100 MHz, $CDCl_3$) δ 172.7, 155.3, 138.2, 128.3, 127.3, 126.5, 122.8, 110.7, 110.6, 83.4, 79.8, 53.9, 52.2, 28.3, 27.9, 25.0, 24.8. ^{11}B NMR (128 MHz, $CDCl_3$) δ 30.6. $[\alpha]_D^{25}$ = +41.1 (c = 0.88, $CHCl_3$). HRMS (ESI) m/z calcd for $C_{23}H_{34}BN_2O_6$ $(M + H)^+$ 445.2504; found 445.2491.

^{1}H-NMR, ^{13}C-NMR and ^{11}B-NMR of Methyl (S)-2-(tert-butoxycarbonyl)amino)-3-(5-(4,4,5,5-tetramethyl-1,3,2-dioxaborolan-2-yl)-1H-indol-3-yl)propanoate (L-4a)

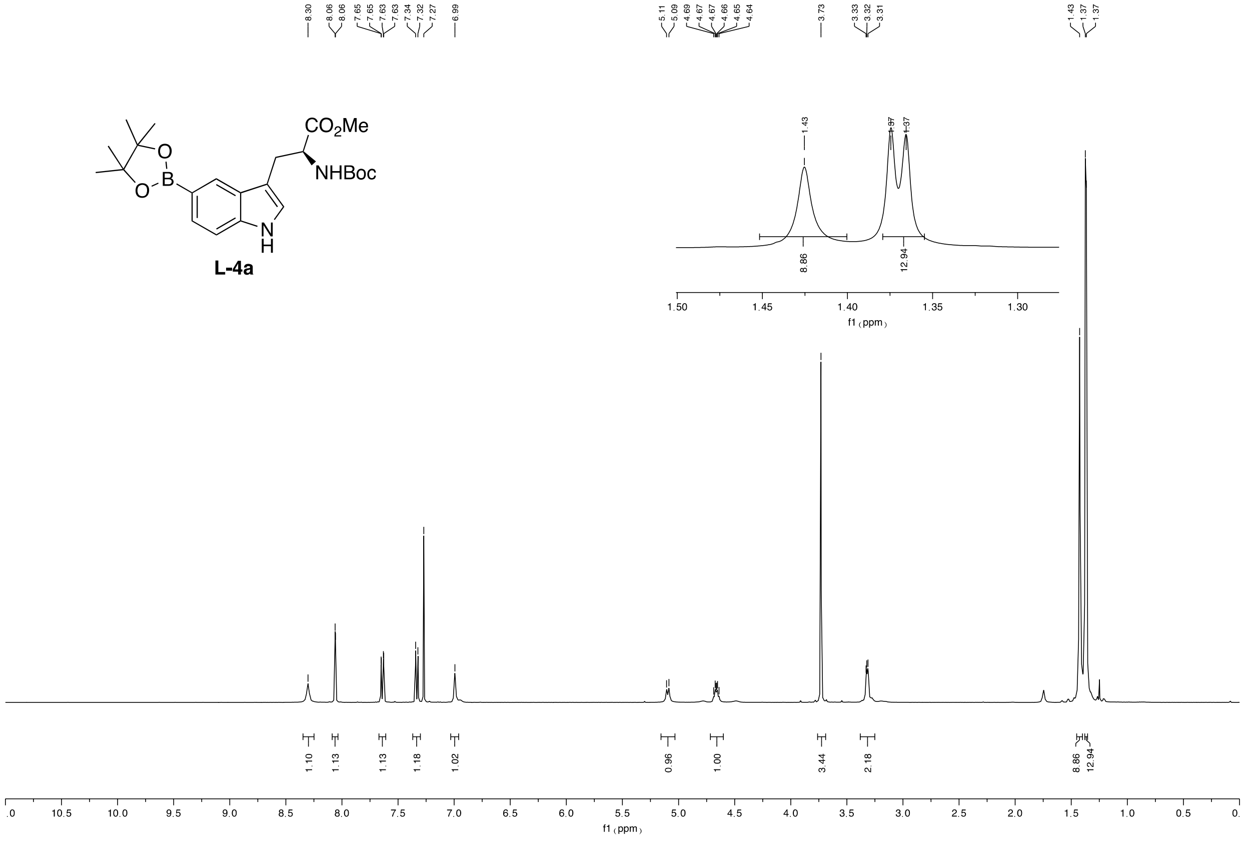
CO2Me
NHBoc
L-4a
8.30
8.06
8.06
7.65
7.65
7.63
7.63
7.34
7.32
7.27
6.99
5.11
5.09
4.69
4.67
4.67
4.66
4.65
4.64
3.73
3.33
3.32
3.31
1.43
1.37
1.37
1.10
1.13
1.13
1.18
1.02
0.96
1.00
3.44
2.18
8.86
12.94
f1 (ppm)

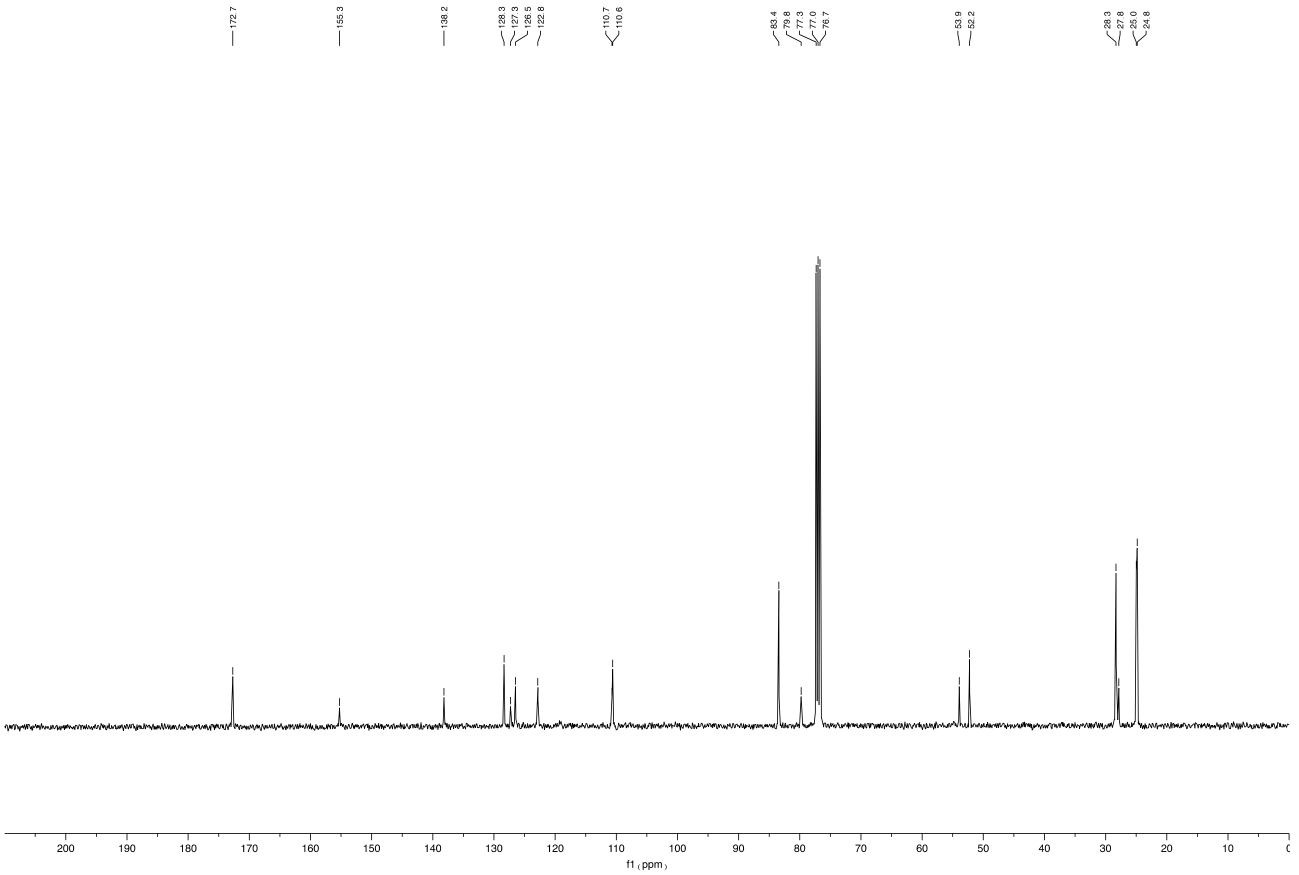
172.7
155.3
138.2
128.3
127.3
126.5
122.8
110.7
110.6
83.4
79.8
77.3
77.0
76.7
53.9
52.2
28.3
27.8
25.0
24.8
f1 (ppm)

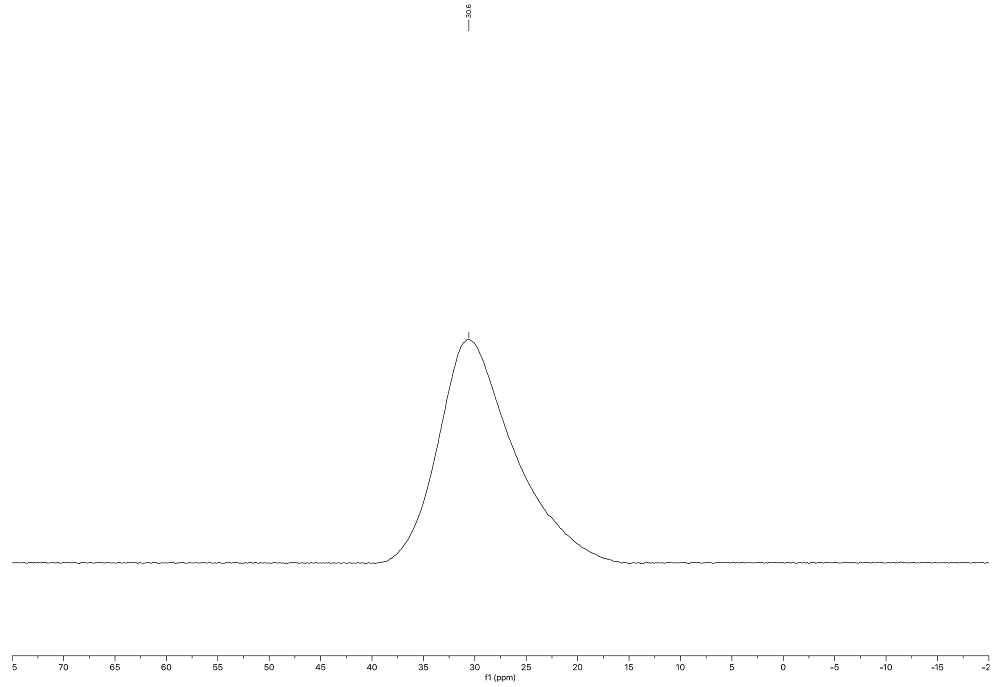

Methyl (R)-2-(tert-butoxycarbonyl)amino)-3-(5-(4,4,5,5-tetramethyl-1,3,2-dioxaborolan-2-yl)-1H-indol-3-yl)propanoate (D-4a) The general procedure was performed between 5-(4,4,5,5-tetramethyl-1,3,2-dioxaborolan-2-yl)-1*H*-indole (**2a**) (364.6 mg, 1.5 mmol) and **D-3** (281 mg, 1 mmol). The crude product was purified by flash chromatography (cyclohexane/EtOAc = 8:2) to yield **D-4a** (244 mg, 55%) as a white solid. The spectroscopic data are identical to those of **L-4a**. $[\alpha]_D^{25}$ = -42 (c = 0.99, $CHCl_3$). HRMS (ESI) m/z calcd for $C_{23}H_{34}BN_2O_6$ $(M + H)^+$ 445.2504; found 445.2491.

Methyl (S)-2-((tert-butoxycarbonyl)amino)-3-(6-(4,4,5,5-tetramethyl-1,3,2-dioxaborolan-2-yl)-1H-indol-3-yl)propanoate (L-4b) The general procedure was performed between 6-(4,4,5,5-tetramethyl-1,3,2-dioxaborolan-2-yl)-1*H*-indole (**2b**) (364.6 mg, 1.5 mmol) and **L-3** (281 mg, 1 mmol). The crude product was purified by flash chromatography (cyclohexane/EtOAc = 8:2) to yield **L-4b** (199 mg, 45%) as a pale-yellow solid. ^{1}H NMR (400 MHz, $CDCl_3$) δ 8.11 (br s, 1H), 7.86 (s, 1H), 7.56 (s, 2H), 7.07 (d, *J* = 2.0 Hz, 1H), 5.07 (d, *J* = 8.0 Hz, 1H), 4.68-4.63 (m, 1H), 3.67 (s, 3H), 3.30 (d, *J* = 4.0 Hz, 2H), 1.43 (s, 9H), 1.37 (s, 12H). ^{13}C NMR (100 MHz, $CDCl_3$) δ 172.6, 155.2, 135.9, 130.1, 125.4, 124.3, 118.2, 118.1, 110.4, 83.6, 79.8, 54.2, 52.2, 28.3, 27.9, 26.9, 24.9. ^{11}B NMR (128 MHz, $CDCl_3$) δ 30.2. $[\alpha]_D^{25}$ = +31.4 (c = 1.21, $CHCl_3$). HRMS (ESI) m/z calcd for $C_{23}H_{34}BN_2O_6$ $(M + H)^+$ 445.2504; found 445.2491.

^{1}H-NMR, ^{13}C-NMR and ^{11}B-NMR of Methyl (S)-2-((tert-butoxycarbonyl)amino)-3-(6-(4,4,5,5-tetramethyl-1,3,2-dioxaborolan-2-yl)-1H-indol-3-yl)propanoate (L-4b)

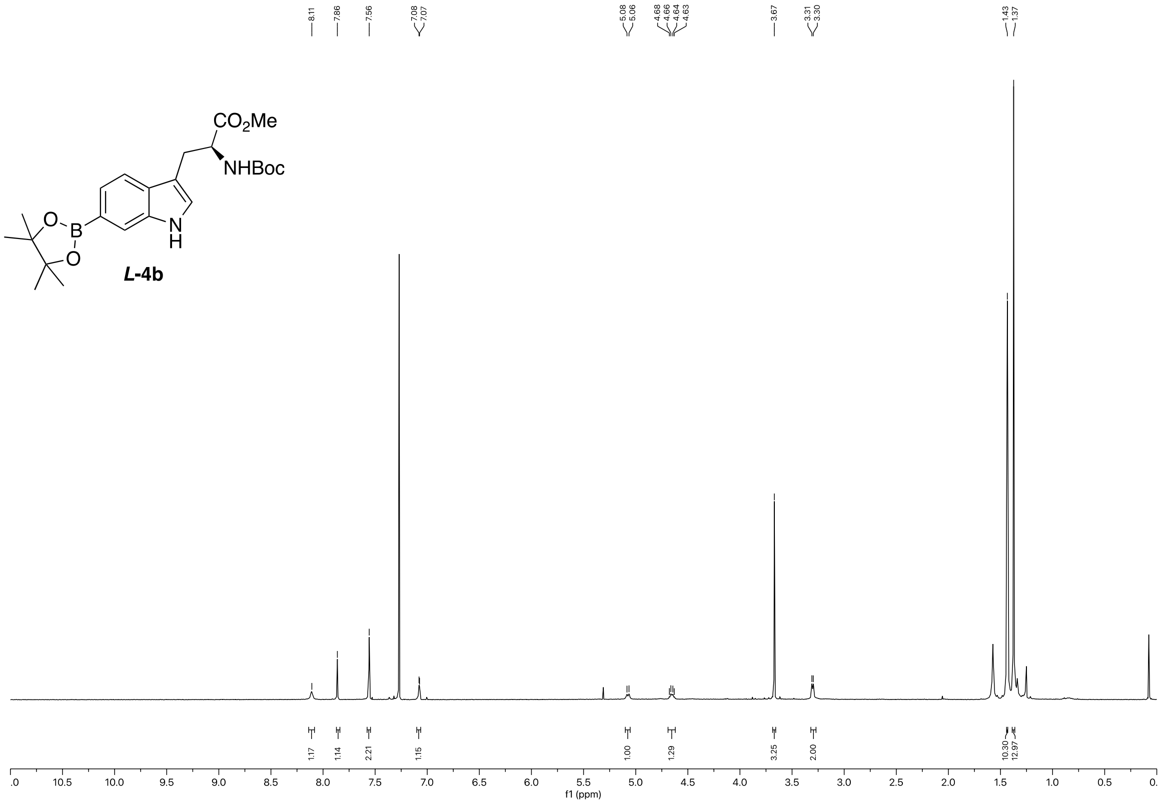
CO2Me
NHBoc
O
B
O
N
H
8.11
7.86
7.56
7.08
7.07
5.08
5.06
4.68
4.66
4.64
4.63
3.67
3.31
3.30
1.43
1.37
1.17
1.14
2.21
1.15
1.00
1.29
3.25
2.00
10.30
12.97
f1 (ppm)

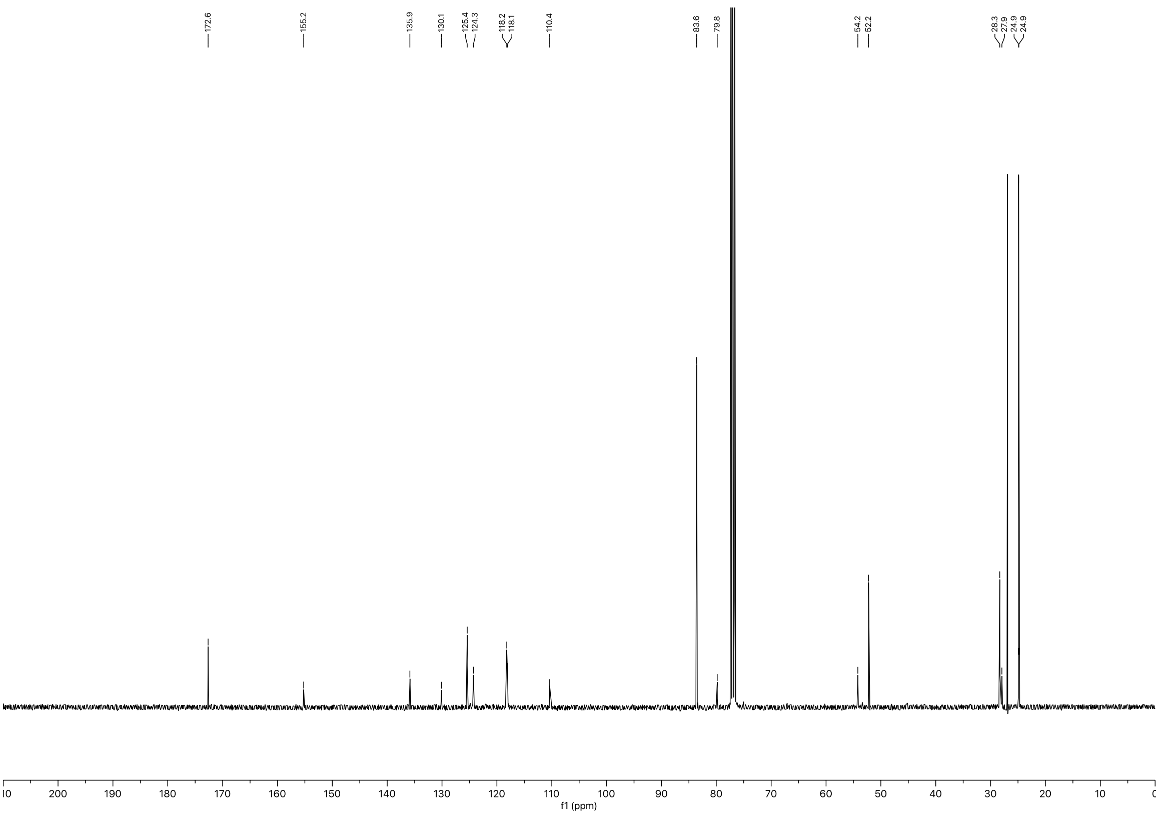
172.6
155.2
135.9
130.1
125.4
124.3
118.2
118.1
110.4
83.6
79.8
54.2
52.2
28.3
27.9
24.9
24.9
f1 (ppm)

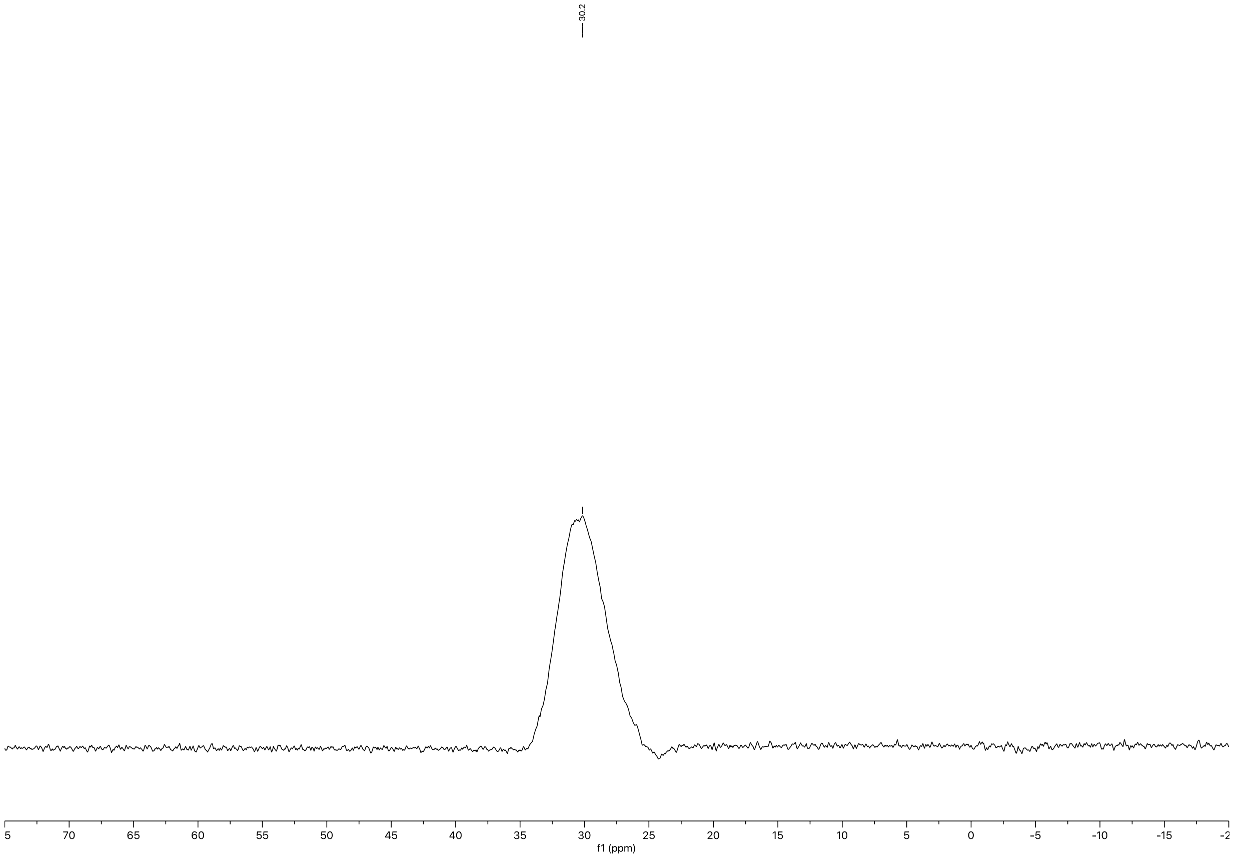

Methyl (R)-2-((tert-butoxycarbonyl)amino)-3-(6-(4,4,5,5-tetramethyl-1,3,2-dioxaborolan-2-yl)-1H-indol-3-yl)propanoate (D-4b) The general procedure was performed between 6-(4,4,5,5-tetramethyl-1,3,2-dioxaborolan-2-yl)-1*H*-indole (**2b**) (364.6 mg, 1.5 mmol) and **D-3** (281 mg, 1 mmol). The crude product was purified by flash chromatography (cyclohexane/EtOAc = 8:2) to yield **D-4b** (178 mg, 40%) as a pale-yellow solid. The spectroscopic data are identical to those of **L-4b**. $[\alpha]_D^{25}$ = -32.1 (c = 0.99, $CHCl_3$). HRMS (ESI) m/z calcd for $C_{23}H_{34}BN_2O_6$ $(M + H)^+$ 445.2504; found 445.2489.

General procedure for deprotection of boronated tryptophans. To a solution of the appropriate boronated tryptophan derivatives (**L-4a,b; D-4a,b**) (1 equiv) was added dropwise HCl 4M in dioxane (10 equiv) and the reaction mixture was stirred at room temperature for 2 h. The solution was diluted with H_2O (6.5 ml x mmol), adjusted at pH = 9 with the addition of NaOH 1 N, and then stirred at room temperature for 2 h. The aqueous phase was washed with diethyl ether (3 x (20 ml x mmol)), concentrated to half volume, and purified by flash chromatography using 70-230 mesh silica gel and eluting with acetonitrile/water 9:1. The fractions containing the product were combined, then the organic solvent was removed in vacuo. The residual water was frozen and lyophilized.

(S)-2-amino-3-(5-borono-1H-indol-3-yl)propanoic acid (L-1a) The general procedure was performed using compound **L-4a** (89 mg, 0.2 mmol). The residue was purified by flash chromatography to give the desired compound **L-1a**, as a pale-yellow solid (44.6 mg, 0.18 mmol, 90%). ^1H NMR (400 MHz, D_2O) δ 8.08 (br s, 1H), 7.57 (dd, *J* = 8.0, 1.0 Hz, 1H), 7.5 (d, *J* = 8. Hz, 1H), 7.28 (s, 1H), 4.00 (dd, *J* = 8.0, 5.0 Hz, 1H), 3.44 (dd, *J* = 15.5, 5.0 Hz, 1H), 3.27 (dd, *J* = 15.5, 8.0 Hz, 1H). $[\alpha]_D^{25}$ = -7.2 (c = 0.87, H_2O). ^{11}B NMR (128 MHz, D_2O) δ 18.8. LC-MS retention time 1.55 min; LRMS m/z [M+H]+: 249.6. HRMS (ESI) m/z calcd for $C_{11}H_{14}BN_2O_4$ $(M + H)^+$ 249.1041; found 249.1038. The spectroscopic data are in accordance with the literature.[4]

^1H-NMR and ^{11}B-NMR of (S)-2-amino-3-(5-borono-1H-indol-3-yl)propanoic acid (L-1a)

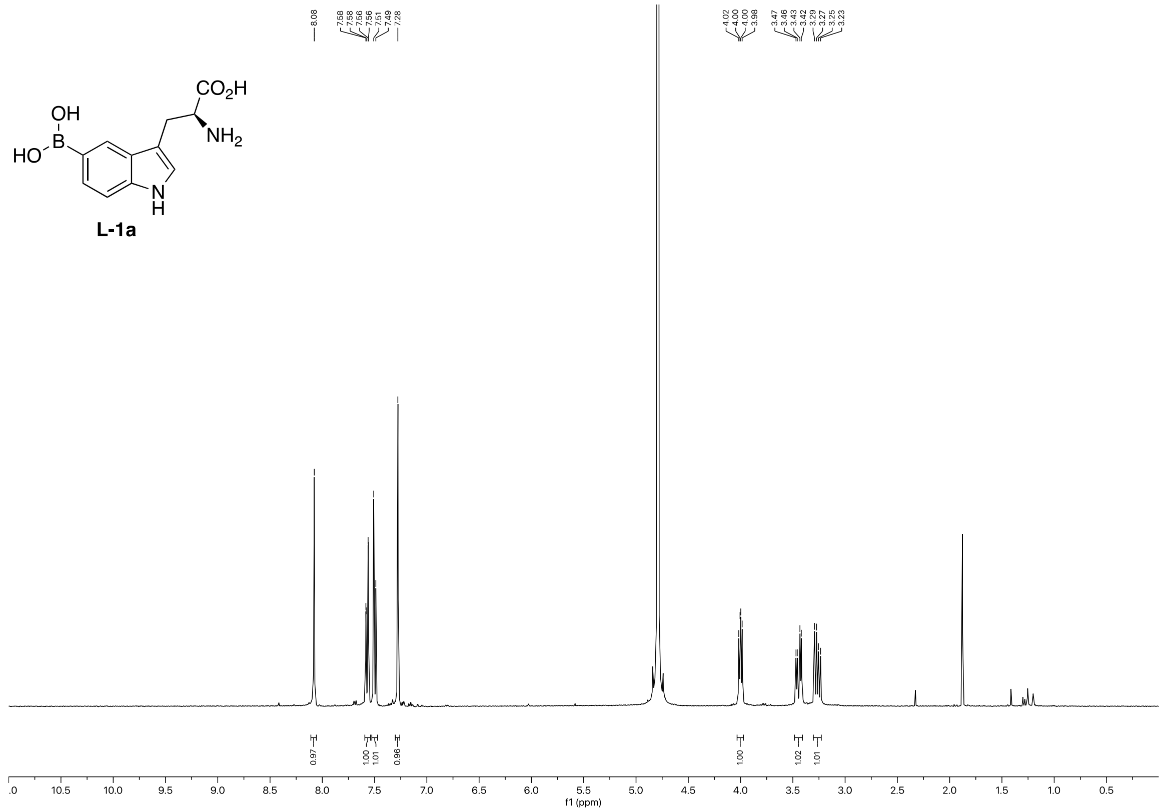
OH
HO
B
CO_2H
NH_2
N
H
L-1a
f1 (ppm)

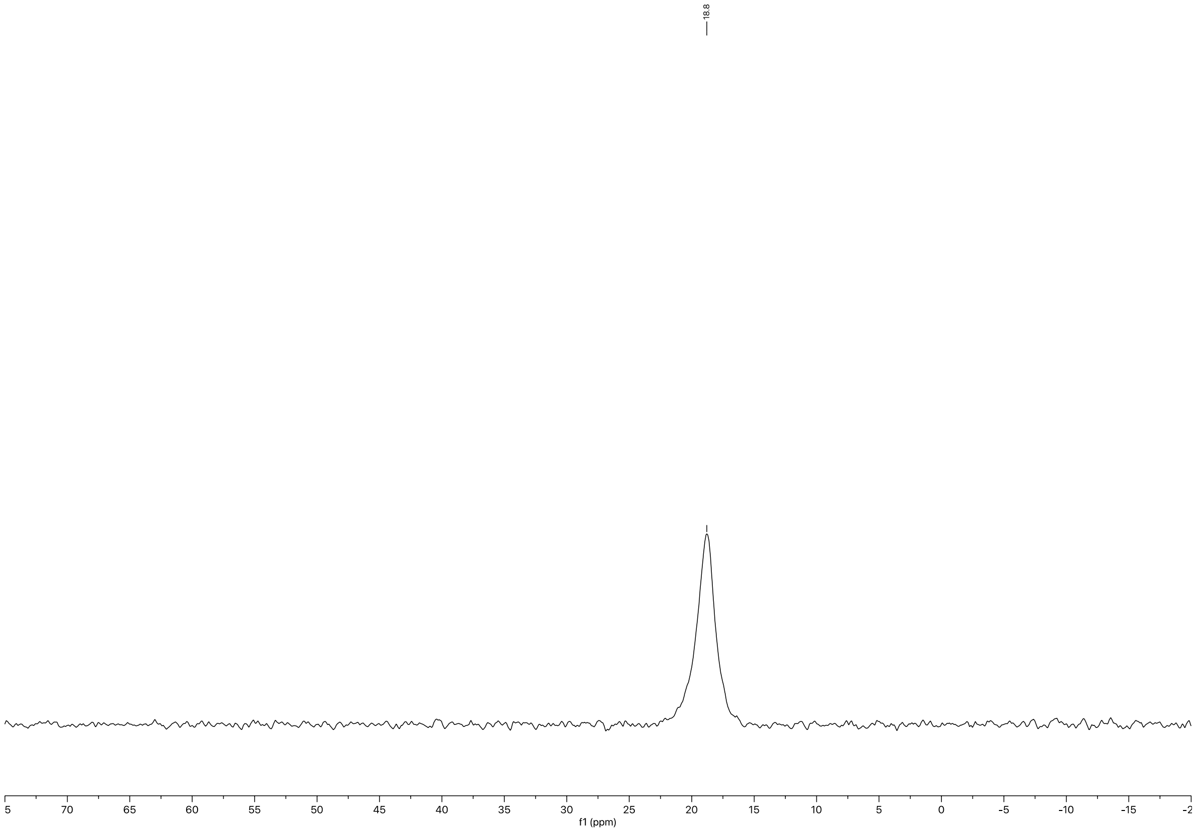
18.8
f1 (ppm)

LC-MS and LRMS of (S)-2-amino-3-(5-borono-1H-indol-3-yl)propanoic acid (L-1a)

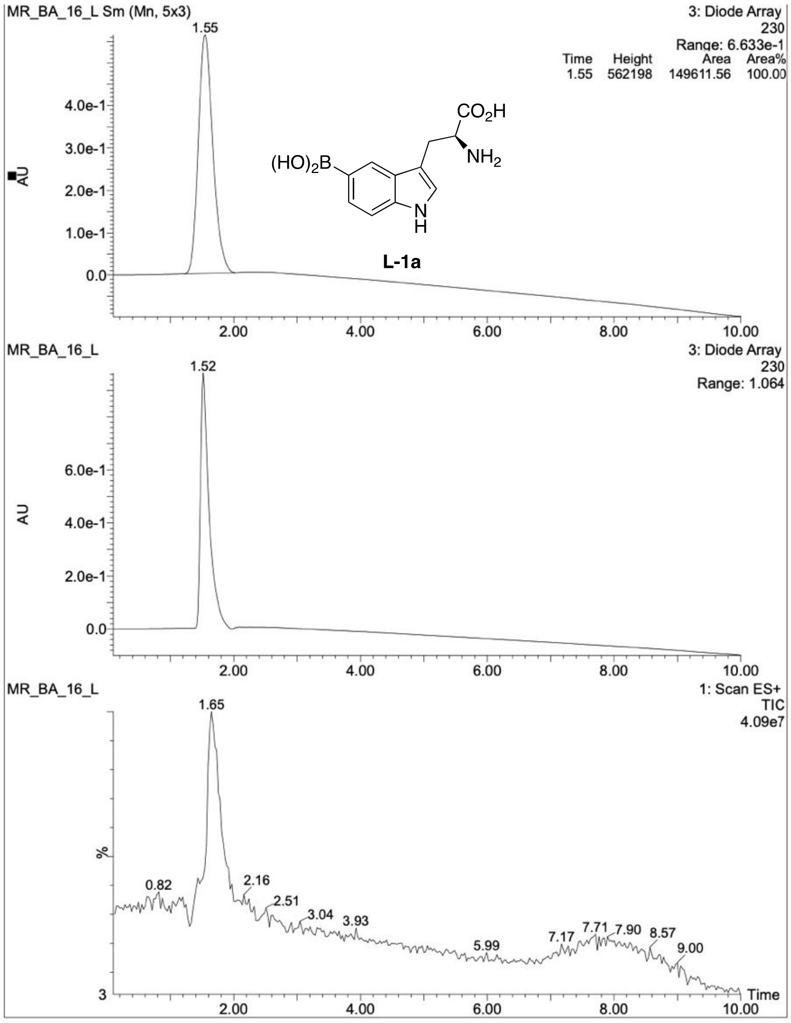

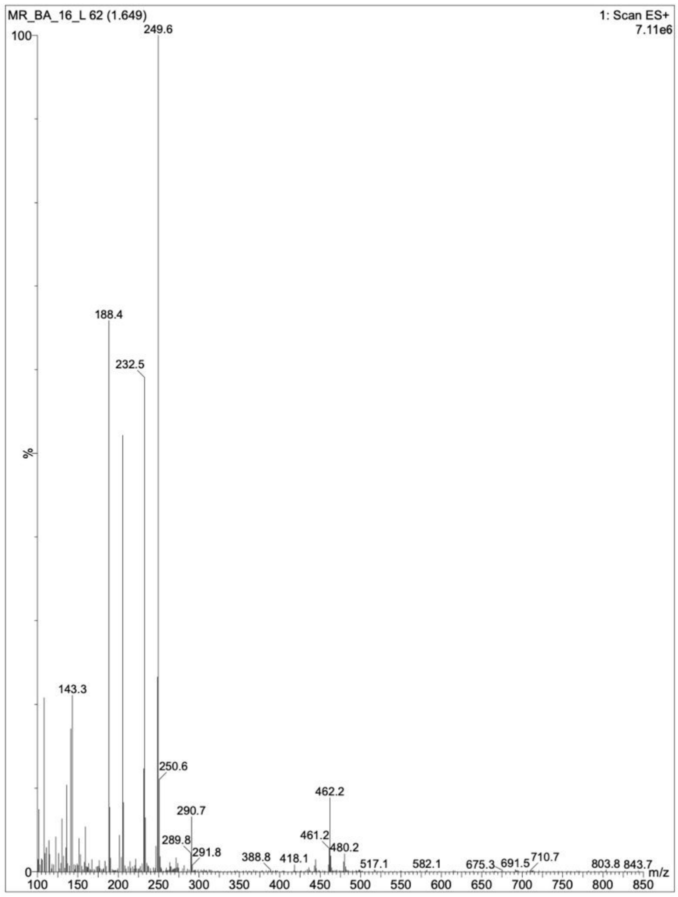

(R)-2-amino-3-(5-borono-1H-indol-3-yl)propanoic acid (D-1a) The general procedure was performed using compound **D-4a** (89 mg, 0.2 mmol). The residue was purified by flash chromatography to give the desired compound **D-1a**, as a pale-yellow solid (42 mg, 0.17 mmol, 85%). The spectroscopic data are identical to those of **L-1a** and in accordance with the literature.[4] $[\alpha]_D^{25}$ = +5.9 (c = 0.45, H_2O). LC-MS retention time 1.55 min; LRMS m/z [M+H]+: 249.5. HRMS (ESI) m/z calcd for $C_{11}H_{14}BN_2O_4$ $(M + H)^+$ 249.1041; found 249.1038.

LC-MS and LRMS of (R)-2-amino-3-(5-borono-1H-indol-3-yl)propanoic acid (D-1a)

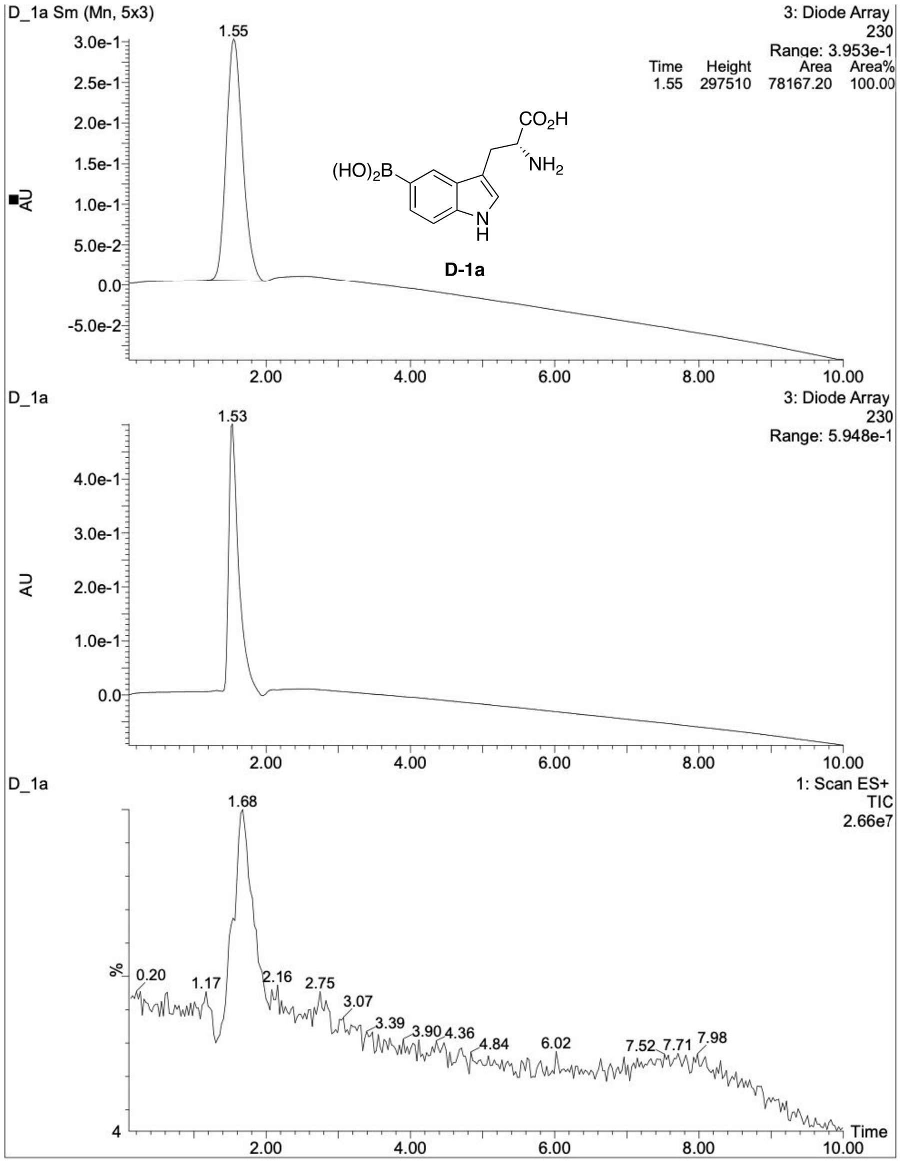
D_1a Sm (Mn, 5x3)
3: Diode Array
230
Range: 3.953e-1
Time Height Area Area%
1.55 297510 78167.20 100.00
1.55
AU
(HO)2B
CO2H
NH2
H
D-1a
D_1a
3: Diode Array
230
Range: 5.948e-1
1.53
AU
D_1a
1: Scan ES+
TIC
2.66e7
1.68
%
Time

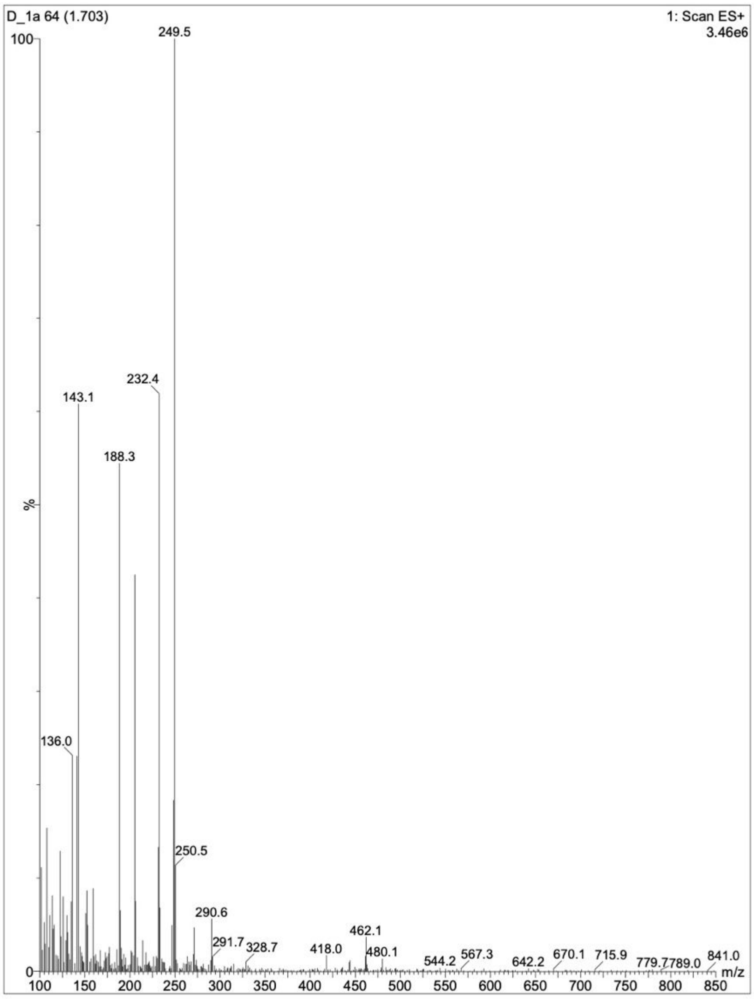

(S)-2-amino-3-(6-borono-1H-indol-3-yl)propanoic acid (L-1b) The general procedure was performed using compound **L-4b** (89 mg, 0.2 mmol). The residue was purified by flash chromatography to give the desired compound **L-1b**, as a pale-yellow solid (39.6 mg, 0.16 mmol, 80%). ^{1}H NMR (400 MHz, D_2O) δ 7.86 (s, 1H), 7.70 (d, *J* = 8.0 Hz, 1H), 7.47 (d, *J* = 8.0 Hz, 1H), 7.33 (s, 1H), 4.00 (dd, *J* = 8.0, 5.0 Hz, 1H), 3.43 (dd, *J* = 15.5, 5.0 Hz, 1H), 3.26 (dd, *J* = 15.5, 8.0 Hz, 1H). ^{11}B NMR (128 MHz, D_2O) δ 18.4. $[\alpha]_D^{25}$ = -9.8 (c = 0.86, H_2O). LC-MS retention time 1.53 min; LRMS m/z [M+H]+: 249.5. HRMS (ESI) m/z calcd for $C_{11}H_{14}BN_2O_4$ $(M + H)^+$ 249.1041; found 249.1038. The spectroscopic data are in accordance with the literature.[5]

^{1}H-NMR and ^{11}B-NMR of (S)-2-amino-3-(6-borono-1H-indol-3-yl)propanoic acid (L-1b)

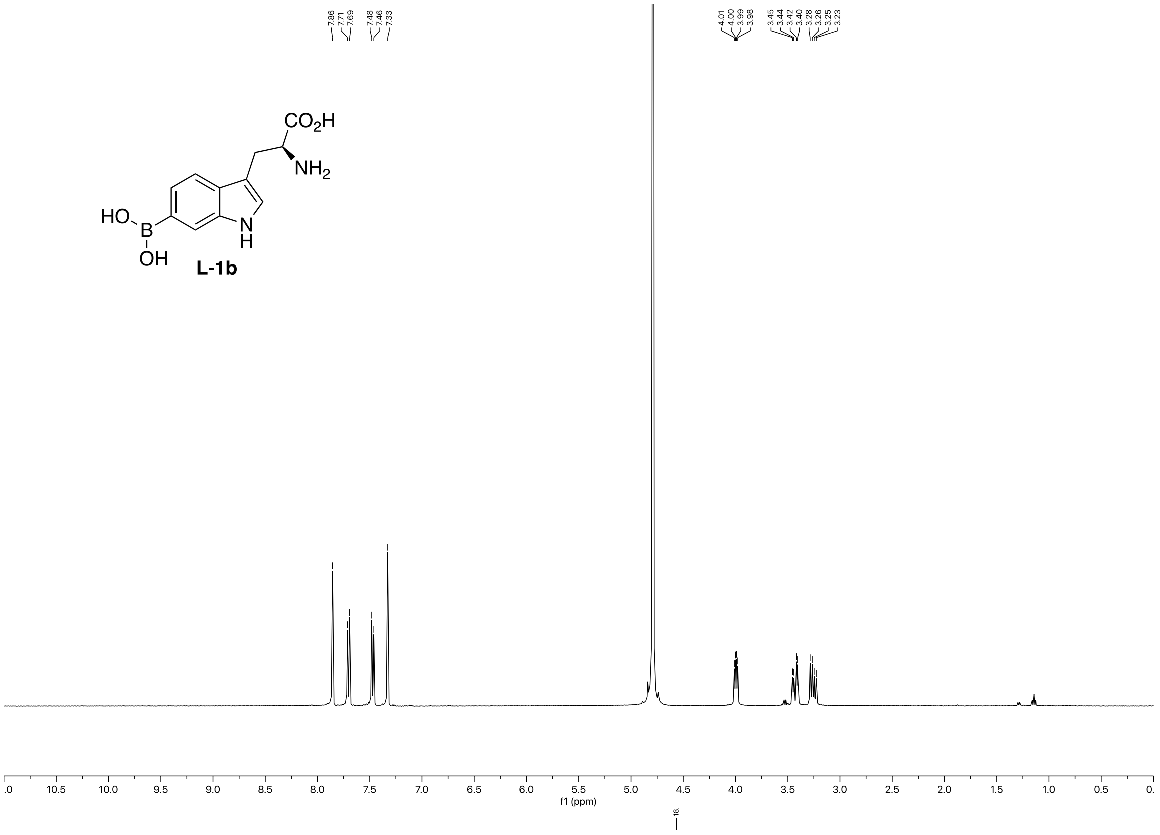

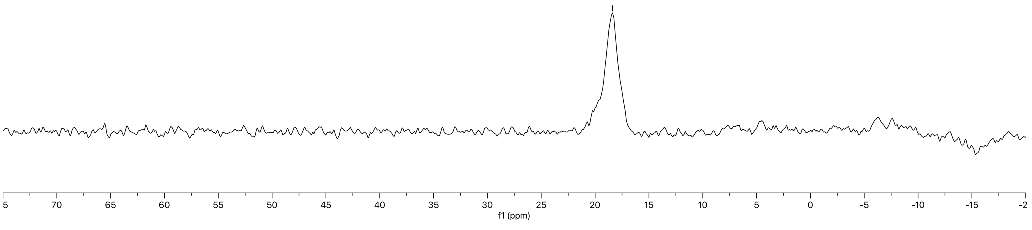

LC-MS and LRMS of (S)-2-amino-3-(6-borono-1H-indol-3-yl)propanoic acid (L-1b)

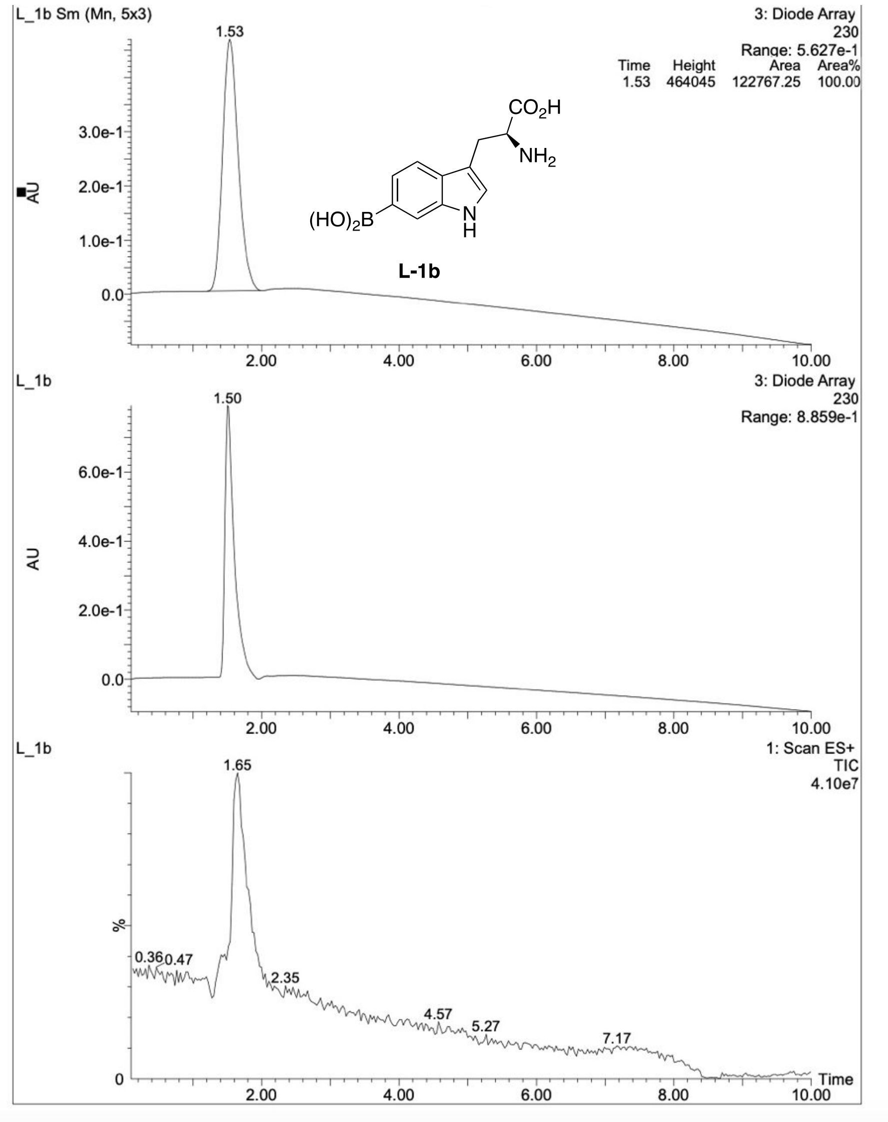
L_1b Sm (Mn, 5x3)
3: Diode Array
230
Range: 5.627e-1
Time Height Area Area%
1.53 464045 122767.25 100.00
1.53
CO2H
NH2
(HO)2B
H
N
L-1b
AU
L_1b
3: Diode Array
230
Range: 8.859e-1
1.50
AU
L_1b
1: Scan ES+
TIC
4.10e7
1.65
%
0.36
0.47
2.35
4.57
5.27
7.17
Time

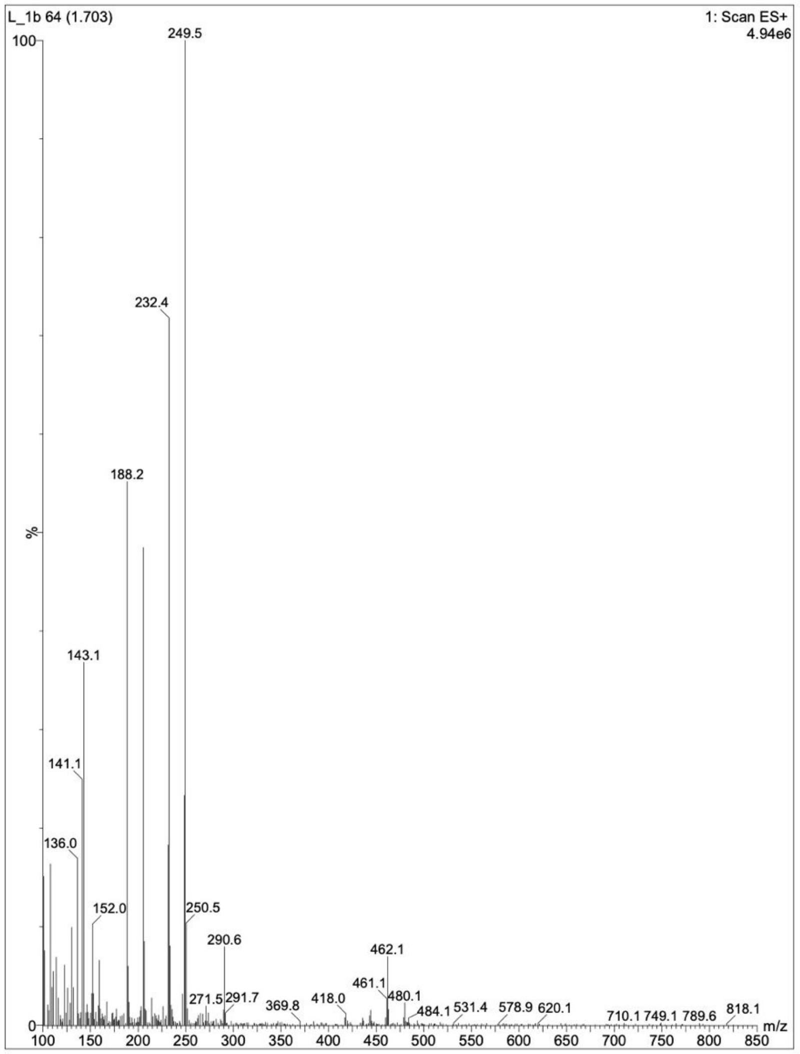

(R)-2-amino-3-(6-borono-1H-indol-3-yl)propanoic acid (D-1b) The general procedure was performed using compound **D-4b** (89 mg, 0.2 mmol). The residue was purified by flash chromatography to give the desired compound **D-1b**, as a pale-yellow solid (42.1 mg, 0.17 mmol, 80%). The spectroscopic data are identical to those of **L-1b** and in accordance with the literature.[5] $[\alpha]_D^{25}$ = +10.1 (c = 0.98, H_2O). LC-MS retention time 1.53 min; LRMS m/z [M+H]+: 249.5. HRMS (ESI) m/z calcd for $C_{11}H_{14}BN_2O_4$ $(M + H)^+$ 249.1041; found 249.1038.

LC-MS and LRMS of (R)-2-amino-3-(6-borono-1H-indol-3-yl)propanoic acid (D-1b)

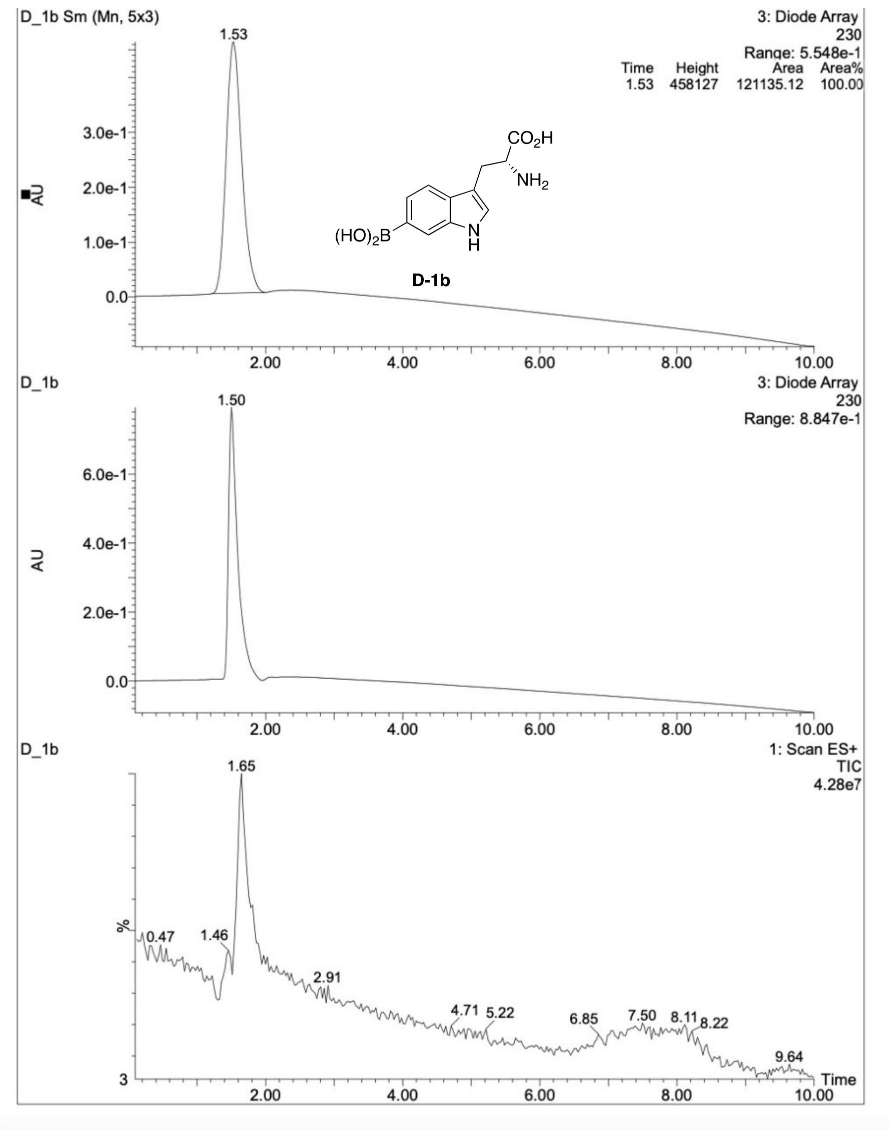
D_1b Sm (Mn, 5x3)
3: Diode Array
230
Range: 5.548e-1
Time Height Area Area%
1.53 458127 121135.12 100.00
1.53
AU
CO2H
NH2
(HO)2B
H
N
D-1b
D_1b
3: Diode Array
230
Range: 8.847e-1
1.50
AU
D_1b
1: Scan ES+
TIC
4.28e7
1.65
%
Time

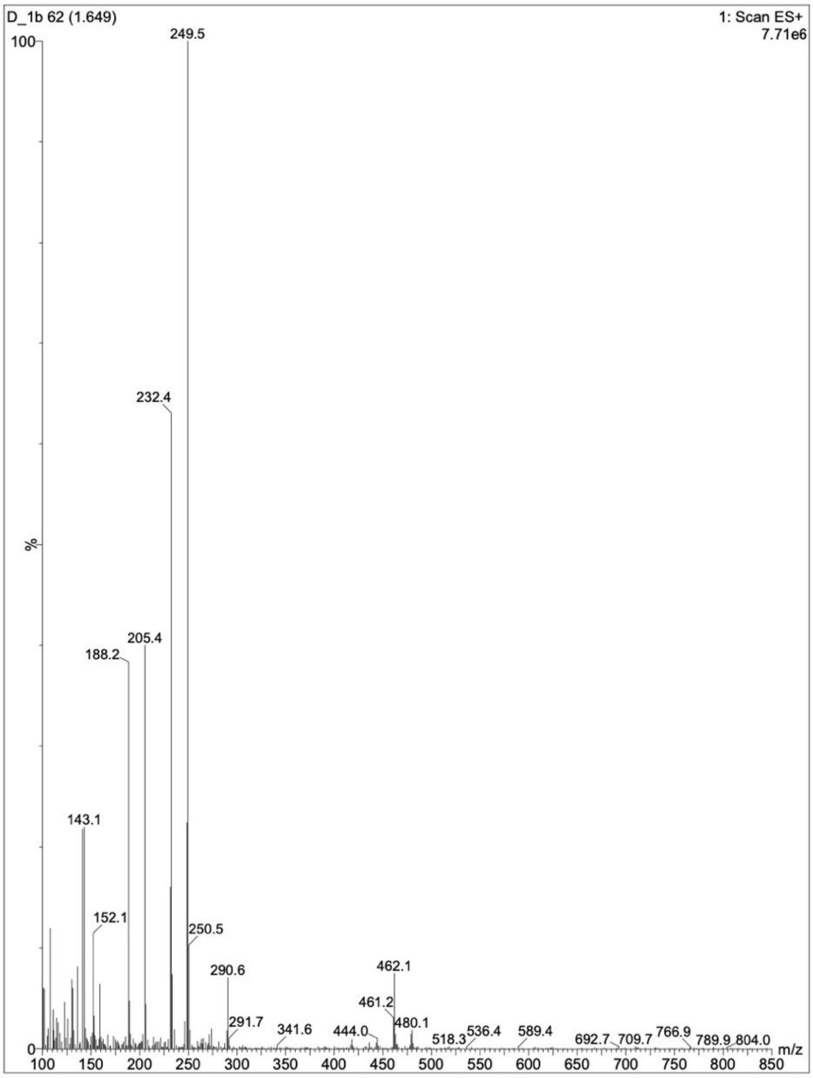

1.2. Biological evaluation

Cell cultures CAL27 squamos cell carcinoma cells (ATCC® CRL-2095™) were purchased from the American Type Culture Collection (ATCC, Manassas, VA, USA). CAL27 cells were cultured in Dulbecco's Modified Eagle Medium (DMEM; Gibco, ThermoFisher Scientific, Waltham, MA, USA) supplemented with 2 mM L-glutamine (ThermoFisher Scientific, Waltham, MA, USA), 10% heat-inactivated fetal bovine serum (Gibco, ThermoFisher Scientific, Waltham, MA, USA), penicillin (50 U/mL)- streptomycin (50 µg/mL) solution (ThermoFisher Scientific, Waltham, MA, USA) at 37 °C in a humidified incubator with 5% CO_2. CAL27 cells were seeded onto 24 well plates at the density of 5 x 10^5 cells/wells. The cells were used for affinity and uptake studies two days after seeding, while the passage number of the cells was in the range of 8-15. The culture medium was removed, and the cells were washed and pre-incubated at 37 °C for 10 min with pre-warmed Hanks' balanced salt solution (HBSS, including 125 mM NaCl, 4.8 mM KCl, 1.2 mM $MgSO_4$, 1.3 mM KH2PO4, 1.3 mM $CaCl_2$, 5.6 mM glucose and 25 mM HEPES adjusted to pH 7.4) before the experiments.

Ability of tryptophan derivatives to inhibit $[^{14}C]$-L-leucine uptake After pre-incubation cells were incubated with 250 µl substrate solutions (concentrations 10 µM, 100 µM and 200 µM, n=3 for each concentration), which included $[^{14}C]$-L-leucine (2.5 µl/ml, 0.1 mCi/mL), for 10 min. Substrate transportation was stopped by adding 500 µl ice-cold HBSS and the cells were washed twice with ice-cold HBSS after which the cells were lysed with 250 µL of 0.1 M NaOH for 60 min at room temperature. The cell lysates were collected into Eppendorf tubes, mixed with 1.0 mL of Emulsifier safe cocktail (Ultima Gold, PerkinElmer, Waltham, MA, USA), and the radioactivity of the lysates was measured by liquid scintillation counting (MicroBeta2 counter, PerkinElmer Waltham, MA, USA). The relative percentage of $[^{14}C]$-L-leucine in cell lysates for different tryptophan substrates was calculated by comparing the radioactivities to controls containing $[^{14}C]$-L-leucine in HBSS.

Concentration-dependent uptake of tryptophan derivatives to CAL27 cells After removing the pre-incubation HBSS, cells were incubated with 250 µL of substrate solutions with concentrations ranging from 10 to 400 µM (n=3 for each concentration) for a 5-minute incubation. Substrate transport was stopped by adding 500 µL of ice-cold HBSS, followed by two washes with ice-cold HBSS. Subsequently, the cells were lysed with 250 µL of a solution containing 75% acetonitrile with 0.1% formic acid and 200 nM labetalol (serving as an internal standard for LC-MS/MS) for 30 minutes. The lysates were collected into Eppendorf tubes and centrifuged at +4 °C at 13,200 rpm for 10 minutes. Following centrifugation, 150 µL from the supernatant was transferred to vials for LC-MS/MS analysis. Protein concentrations were determined in the cell lysates, obtained from the same plates as the samples (lysed with 0.1 M NaOH), using the Bio-Rad Protein Assay, based on the Bradford dye-binding method. Absorbance at 595 nm was measured with a multiplate reader (EnVision, Perkin Elmer, Inc., Waltham, MA, USA). Bovine serum albumin (BSA) was employed as the standard for protein quantification. The results were calculated as pmol of studied compounds per mg of protein.

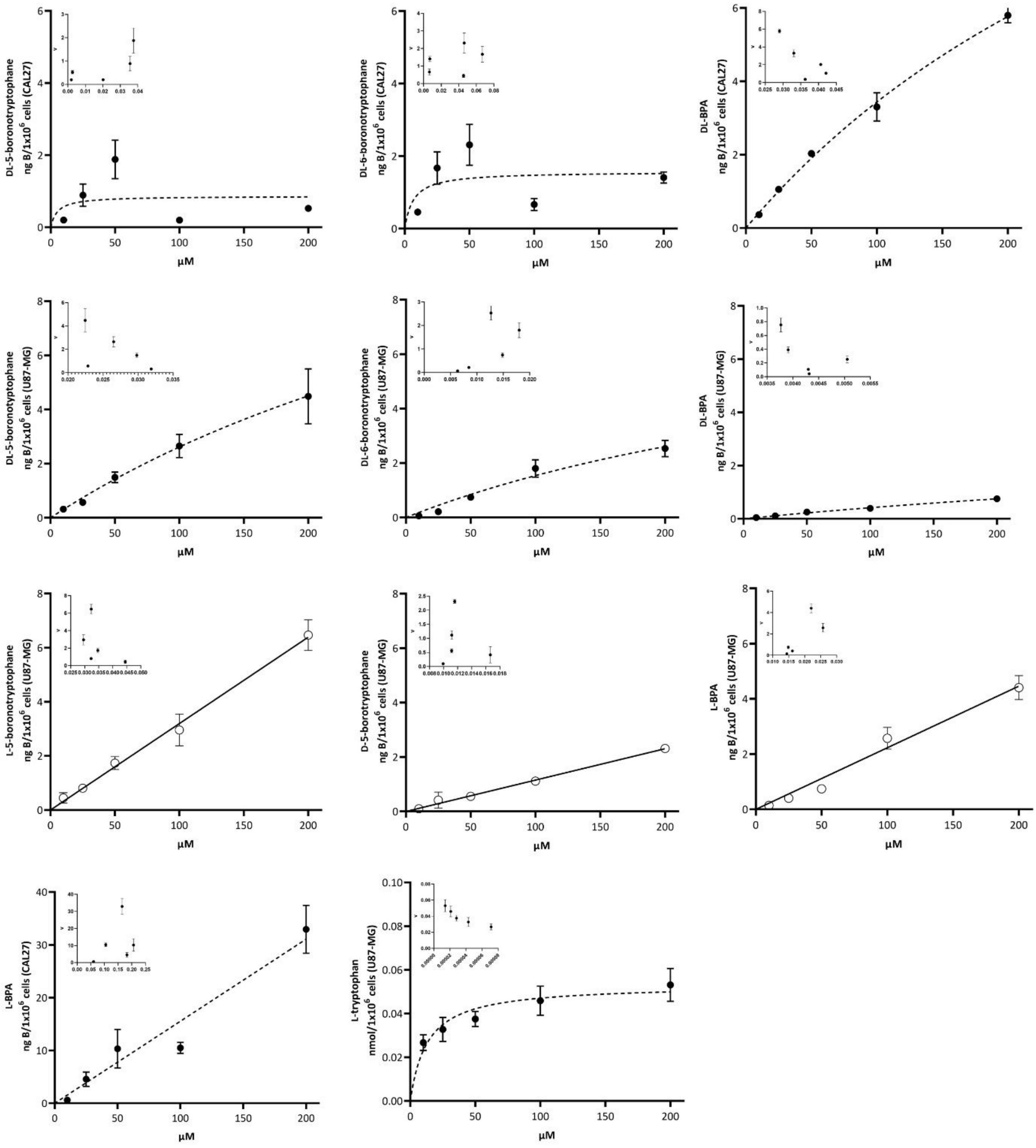

Figure S1. Uptake profiles of DL-5-boronotryptophan, DL-6-boronotryptophan, 5-L-boronotryptophan, 5-D-boronotryptophan, DL-BPA, and L-BPA in CAL27 and U87-MG cells. L-tryptophan uptake is also shown in U87-MG cells. Insets show Eadie-Hofstee plots.

Table S1. A) Average (±SD) concentration of each compound taken up by cells after incubation with varying concentrations of the test compounds, presented as ng B/1×10^6 cells and ng B/mg protein**. B)** Michaelis-Menten kinetic parameters for the studied compounds, presented as ng B/1×10^6 cells and ng B/mg protein.

A.

µM	DL-5-boronotryptophane	DL-6-boronotryptophane	L-5-boronotryptophane	D-5-boronotryptophane	DL-BPA	L-BPA	L-tryptophan**
10	0.32±0.07	0.06±0.04	0.44±0.19	0.10±0.03	0.04±0.01	0.14±0.02	0.027±0.004
25	0.57±0.07	0.21±0.02	0.81±0.13	0.42±0.29	0.11±0.01	0.40±0.02	0.033±0.006
50	1.49±0.20	0.74±0.09	1.73±0.24	0.56±0.06	0.25±0.05	0.74±0.13	0.038±0.003
100	2.65±0.43	1.80±0.32	2.96±0.58	1.12±0.15	0.39±0.04	2.57±0.39	0.046±0.007
200	4.49±1.01	2.53±0.30	6.47±0.57	2.32±0.06	0.75±0.10	4.41±0.43	0.053±0.0.8

ng B/1x10^6 cells (CAL27)

µM	DL-5-boronotryptophane	DL-6-boronotryptophane	L-5-boronotryptophane	D-5-boronotryptophane	DL-BPA	L-BPA	L-tryptophan
10	0.20±0.01	0.45±0.09	N/M	N/M	0.36±0.02	0.60±0.48	N/M
25	0.89±0.31	1.67±0.45	N/M	N/M	1.05±0.04	4.56±1.36	N/M
50	1.88±0.54	2.31±0.56	N/M	N/M	2.02±0.02	10.33±3.65	N/M
100	0.20±0.05	0.66±0.16	N/M	N/M	3.30±0.38	10.49±1.03	N/M
200	0.52±0.07	1.41±0.15	N/M	N/M	5.80±0.20	32.93±4.54	N/M

ng B/mg of protein (U87)

µM	DL-5-boronotryptophane	DL-6-boronotryptophane	L-5-boronotryptophane	D-5-boronotryptophane	DL-BPA	L-BPA	L-tryptophan**
10	3.06±0.70	0.54±0.37	3.52±1.51	0.80±0.22	0.68±0.08	0.81±0.13	0.37±0.05
25	5.49±0.66	1.82±0.13	6.38±1.04	3.34±2.35	1.70±0.10	2.28±0.09	0.46±0.08
50	14.32±1.88	6.32±0.79	13.70±1.88	4.47±0.51	3.99±0.84	4.19±0.73	0.52±0.05
100	25.47±4.12	15.48±2.72	23.38±4.62	8.96±1.23	6.18±0.65	14.53±2.23	0.64±0.09
200	43.13±9.75	21.78±2.59	51.11±4.48	18.60±0.50	11.19±1.60	24.89±2.44	0.74±0.10

ng B/mg of protein (CAL27)

µM	DL-5-boronotryptophane	DL-6-boronotryptophane	L-5-boronotryptophane	D-5-boronotryptophane	DL-BPA	L-BPA	L-tryptophan
10	1.21±0.09	2.49±0.51	N/M	N/M	2.48±0.13	4.49±3.54	N/M
25	5.32±1.85	9.15±2.45	N/M	N/M	7.20±0.28	33.96±10.16	N/M
50	11.24±3.20	12.66±3.08	N/M	N/M	13.89±0.11	76.96±27.16	N/M
100	1.19±0.30	3.63±0.89	N/M	N/M	22.60±2.63	78.12±7.69	N/M
200	3.14±0.41	7.71±0.85	N/M	N/M	39.67±1.39	245.28±33.79	N/M

**result presented as nmol/1x10^6 cells or nmol/mg of protein

B.

Compound	CAL27				U87-MG			
	V_{max} (ng B/1x10^6 cells)	K_m (µM)	V_{max} (ng B/mg of protein)	K_m (µM)	V_{max} (ng B/1x10^6)	K_m (µM)	V_{max} (ng B/mg of protein)	K_m (µM)
DL-5-boronotryptophane	0.86±0.37	5.25±1.9	5.16±2.2	5.25±4.1	16.15±7.8	517.2±180.4	155.1±75.38	517.2±180.4
DL-6-boronotryptophane	1.57±0.98	6.66±2.34	8.59±5.40	6.66±3.7	8.52±4.56	454.1±180.4	73.28±39.26	454.1±180.4
L-5-boronotryptophane	N/M	N/M	N/M	N/M	N/A	N/A	N/A	N/A
D-5-boronotryptophane	N/M	N/M	N/M	N/M	N/A	N/A	N/A	N/A
DL-BPA	17.89±13.57	420.7±283.7	122.4±92.8	420.7±283.7	3.812±1.74	294.3	60.25±27.64	818.1±294.3
L-BPA	N/A	N/A	N/A	N/A	N/A	N/A	N/A	N/A
L-tryptophan	N/M	N/M	N/M	N/M	0.05±3.21**	13.05±6.19	0.74±13.3**	13.05±6.19

*N/M not measured

*N/A not available

** result presented as nmol/1x10^6 cells or nmol/mg of protein

1.3. Analytical methods

LC-MS/MS analysis The samples were analyzed with an Agilent 1200 series Rapid Resolution Liquid Chromatography System (Agilent Technologies, Waldbronn, Germany) coupled with an Agilent 6410 Triple Quadrupole (LC-MS/MS) with electrospray ionization (ESI) (Agilent Technologies, Palo Alto, CA, USA). The samples (5 µL) were injected into the reversed-phase HPLC column (Zorbax Eclipse XDB-C18 Rapid Resolution 4.6x50 mm, 1.8 µm, Agilent Technologies, Palo Alto, CA, USA). The aqueous mobile phase was 0.1% formic acid in water (A), while the organic mobile phase was 0.1% formic acid in acetonitrile (B). The column temperature was +40 °C, and the mobile phase flow rate of 0.3 mL/min was used for all the studied compounds. The following gradient was used: 0-1.5 min: 20% -> 90% B, 1.5-5 min: 90% B, 5-5.5 min: 90% -> 20% B, 5.5-9 min: 20% B. The following instrument parameters were used: drying gas flow rate 8 L/min with +300 °C temperature, 40 psi nebulizer pressure, and 4 kV capillary voltage. Detection was done by using multiple reaction monitoring (MRM) in a positive ionization mode (ESI+) with fragmentor voltage 100 V for L-5-boronotryptophane, D-5-boronotryptophane, and DL-5-boronotryptophane; 90 V for DL-6-boronotryptophane; 100 V for BPA; and 70 V for labetalol. Following transitions were recorded with collision energies in brackets: m/z 249 -> 232 (9 V)/188 (10 V)/146 (20 V) for L-5-boronotryptophane, D-5-boronotryptophane and DL-5-boronotryptophane; m/z 249 -> 232 (10 V)/190 (15 V)/146 (20 V) for DL-6-boronotryptophane; m/z 210 -> 146 (19 V)/120 (19 V)/103 (30 V) for BPA; and m/z 329 -> 294 (10 V)/162 (10 V) for labetalol.

The lower limit of quantification (LLOQ) for all studied compounds was 5 nM. These LC-MS/MS methods were documented to be highly selective, accurate (RSD < 15%), and precise (RSD < 15%) over the range of 5 – 5 000 nM, with acceptable linearity ($R^2 > 0.997$).

Statistical analysis All statistical analyses were performed using GraphPad Prism 9.2.0 software (GraphPad Software, San Diego, CA, USA). Statistical differences between groups were tested using one-way ANOVA, followed by Tukey's multiple comparison test, and presented as the mean ± SD.

1.4. Molecular modeling

Protein preparation Selected experimental cryo-EM structure of the human LAT1 transporter was obtained from the RCSB database (PDBID: 7DSQ). From the downloaded PDB, LAT1 structures were extracted, and cryo-EM additives were removed, while solvent molecules and 3,5-diiodo-L-tyrosine ligand bound to the transmembrane channel were kept. The docking templates were preprocessed, protonation states were optimized and finally minimized (heavy atoms constraint 0.3 Å) with the Protein preparation wizard of Schrodinger Maestro suite 2022-2 and OPLS4 force field (Schrödinger Release 2022-2: Protein preparation wizard, Schrödinger, LLC, New York, NY, 2022).

Ligand preparation L-isomeric structures of small molecule ligands were sketched using Maestro-GUI and the OPLS4 force field parametrization was carried out with the LigPrep module of Schrodinger suite Schrödinger Maestro software package (Schrödinger Release 2022-2: Schrödinger, LLC, New York, NY, 2022).

Molecular docking Induced fit docking allowing partial protein flexibility was employed, where the center of the grid box was positioned to the center of the Cryo-EM ligand taken from the complex refinement described above. The unconstrained ligand docking was performed using standard Prime mapping of the Induced Fit protocol of the Schrodinger suite with the default SP setting of Glide docking resulting in 20 docking poses for visual inspection.

Molecular dynamics simulations Molecular dynamics simulations were carried out using Schrödinger Desmond (Schrödinger Release 2023-2: Desmond Molecular Dynamics System, D. E. Shaw Research, Maestro-Desmond Interoperability Tools, Schrödinger, New York, NY, 2023). The orientation of the LAT1 inside the membrane was defined using the OPM database at the PPM web server. In Desmond system builder, the orthorhombic periodic systems, including the POPC membrane, were created and solvated using TIP3P waters. Systems were further neutralized, including 0.1 M NaCl buffer. At the beginning of the simulation, the system was subjected to the default relaxation protocol of Desmond and heated up to a simulation temperature of 300K. Five unconstrained replicated simulations (randomized seed) up to a length of 1.0 µs were run using NPT protocol at a temperature of 300 K, pressure of 1.01325 bar, Noe-Hoover thermostat, and timestep of 2 fs. Simulations of 5 replicated trajectories containing 5000 snapshots altogether were combined, aligned, and converted to Gromacs xtc-format, after which they were subjected to simulation interactions analysis. Finally, essential dynamics analysis was run using the Schrödinger covariance analysis tool (trj_essential_dynamics.py).

Graphical presentations Schrodinger Maestro 2023-2 was used for visual inspection of docking poses and Graphical illustrations were generated using PyMol v.2.3.0.

2. Characterization of CAL27 line

2.1. LAT1 function

To evaluate the functionality of LAT1 in CAL27 cells, we conducted a comprehensive analysis of their [^{14}C]-L-leucine uptake under various experimental conditions. CAL27 cells, ranging from passages 4 to 10, were seeded at a density of 1 x 10^5 cells per well in 24-well plates. These characterization studies were carried out two days after cell seeding.

Prior to commencing the experiments, the cells were pre-washed with pre-warmed HBSS (Hank's Balanced Salt Solution) and pre-incubated for 10 minutes. To investigate the time-dependent uptake of [^{14}C]-L-leucine, the cells were subjected to incubation for 10 different time intervals, ranging from 0.5 to 60 minutes (n=4). This incubation was performed with 250 µL of HBSS containing 0.76 µM (0.1 mCi/mL) of [^{14}C]-L-leucine. The reaction was stopped by the addition of 500 µL of ice-cold HBSS after incubation, followed by two washes with 500 µL of ice-cold HBSS. The cells were subsequently lysed using 250 µL of 0.1 M NaOH at room temperature for 60 minutes. The lysate was then combined with 1.0 mL of Emulsifier-safe cocktail (PerkinElmer, Waltham, MA, USA), and radioactivity was quantified using a liquid scintillation counter (MicroBeta2 counter, PerkinElmer, Waltham, MA, USA). The optimal incubation time was determined based on the linear range of the time-dependent uptake curve, and a 5-minute incubation time was selected for further investigations into substrate dependency, Na^+-free conditions, the impact of a specific LAT1 inhibitor, and variations in pH (Fig. S1).

To demonstrate the LAT1 expression levels in CAL27 cells, the localization of the LAT1/4F2hc complex was confirmed with immunofluorescence staining as well as quantifying the content of LAT1 and 4F2hc protein. In immunofluorescence staining, the cells were cultured in 6-well plates at a density of 200 000 cells/well, with 2-3 cover glasses (VWR, San Francisco, CA) on the bottom of the wells. Two days after seeding, the cells were chilled at +4 °C for 2 h, washed with chilled 1xPBS 3 mL, and immediately fixed with 1.5 mL of 100% MeOH at -20 °C overnight. The cells were stained on the next day after fixing. Shortly, the cells were washed with 0.1% BSA (2x3 mL), blocked, and permeabilized with 1.5 mL of 1% BSA with 0.1% Triton X-100 at RT for 45 minutes. The cells were incubated with primary antibody dilutions 1:100 (in 1% BSA with 0.1% Triton X-100) on cover glasses at RT for 1.5 h (LAT1#5347 for LAT1, Cell Signaling Technology, Danvers, Massachusetts, USA and AM33318PU-T for 4F2hc, OriGene Technologies, Inc., Rockville, USA). After primary antibody incubation, the cells were washed twice with 0.1% BSA, secondary antibody dilution was prepared 1:750 (in 1% BSA with 0.1% Triton X-100) (Alexa Fluor 594 for LAT1, Alexa Fluor 488 for 4F2hc, Thermo Fisher Scientific, Inc., Bleiswijk, Netherlands) and the cells were incubated at humidified chamber for 1h in the dark. After the incubations, the cells were washed with 1xPBS, coverslips were placed on polylysine microscope slides with ProLong™ Glass Antifade Mountant with NucBlue™ Stain (ThermoFisher Scientific, Waltham, MA, USA), and imaged with the Zeiss Axio Imager M2 coupled with Apotome and AxioCam MR M3 camera (Carl Zeiss Microimaging GmbH, Jena, Gottingen, Germany) (Fig. S2).

2.2. Fluorescence microscopy imaging

To quantify the LAT1 protein amount from crude membrane fractions of CAL27 cells, the sample preparation was performed as described previously.[6] Shortly, the Membrane Protein Extraction Kit (BioVision Incorporated, Milpitas, CA, USA) was used for the extraction of crude membrane fractions from the cell pellet according to the manufacturer's protocol. The protein concentration was measured using a Bio-Rad Protein Assay (EnVision, PerkinElmer, Inc., Waltham, MA, USA), and 50 µg of protein from each sample (n = 3) was taken for further analysis. Further sample preparation was performed as described earlier[6,7] and the absolute LAT1 quantity was determined by an LC-MS/MS-SRM setup. In more

detail, the quantification of LAT1 and the membrane marker Na^+/K^+-ATPase was based on three selected reaction monitoring (SRM) transitions of precursor and product ions from both the light and heavy peptide chains, as previously described. A total of 20 µL of the digested peptides (10 µg) was injected into an Agilent 1290 LC system coupled with an Agilent 6495 triple quadrupole mass spectrometer with an electrospray ionization source operated in the positive mode (Agilent Technologies, Santa Clara, CA, USA). Initially, the peptides were separated on a ^{2}.1 × 250 mm, 2.7 µm column (Agilent Technologies, Santa Clara, CA, USA) and eluted by a gradient of 0.1% formic acid in water (A) and acetonitrile (B). A constant flow rate of 0.3 mL/min was utilized, and the gradient was shifted in the following way: 2–7% B for 2 min, followed by 7–30% B for 48 min, 30–45% B for 3 min, and 45–80% B for 2.5 min before re-equilibrating the column for 4.5 min. The data were acquired using the Agilent MassHunter Workstation Acquisition and processed using the Skyline software 20.1. The LAT1 and Na^+/K^+- ATPase proteins were quantified based on the ratio between the light and heavy peptides (Fig. S2).

2.3. Western blot

Western blot was performed as described earlier [8]. Briefly, the cells were lysed using RIPA lysis buffer (Sigma-Aldrich) supplemented with phosphatase and protease inhibitors (Roche, Basel, Switzerland). Protein concentration from the lysates was measured with the BCA protein assay kit according to manufacturer's instructions (Thermo Fisher Scientific). Proteins were transferred to polyvinylidene fluoride (PVDF) membrane using Trans-Blot Turbo equipment (Bio-Rad). Blocking of the membrane was performed with 5% BSA in Tris-buffered saline and Tween (TBST). LAT1 Rabbit primary antibody (5347S, Cell Signaling Technology) was diluted to 1:1000 in 1% BSA in TBST and the membrane was incubated overnight. Anti-α-Tubulin Mouse monoclonal antibody (T6199, Sigma-Aldrich) was used as loading control, diluted to 1:1000 in 1% BSA in TBST. Anti-Rabbit IgG secondary antibody (65-6120, Invitrogen) and Anti-Mouse IgG secondary antibody (62-6520, Invitrogen) were diluted to 1:10 000 in 1% BSA in TBST. Membrane was washed with TBST washing buffer. Detection reagents for chemiluminescence were obtained from Merck-Millipore (Burlington, MA, USA). CHEMIDOC (Bio-Rad) and Adobe Photoshop and Illustrator CC (Adobe Inc., San Jose, CA, USA) were used for result visualization.

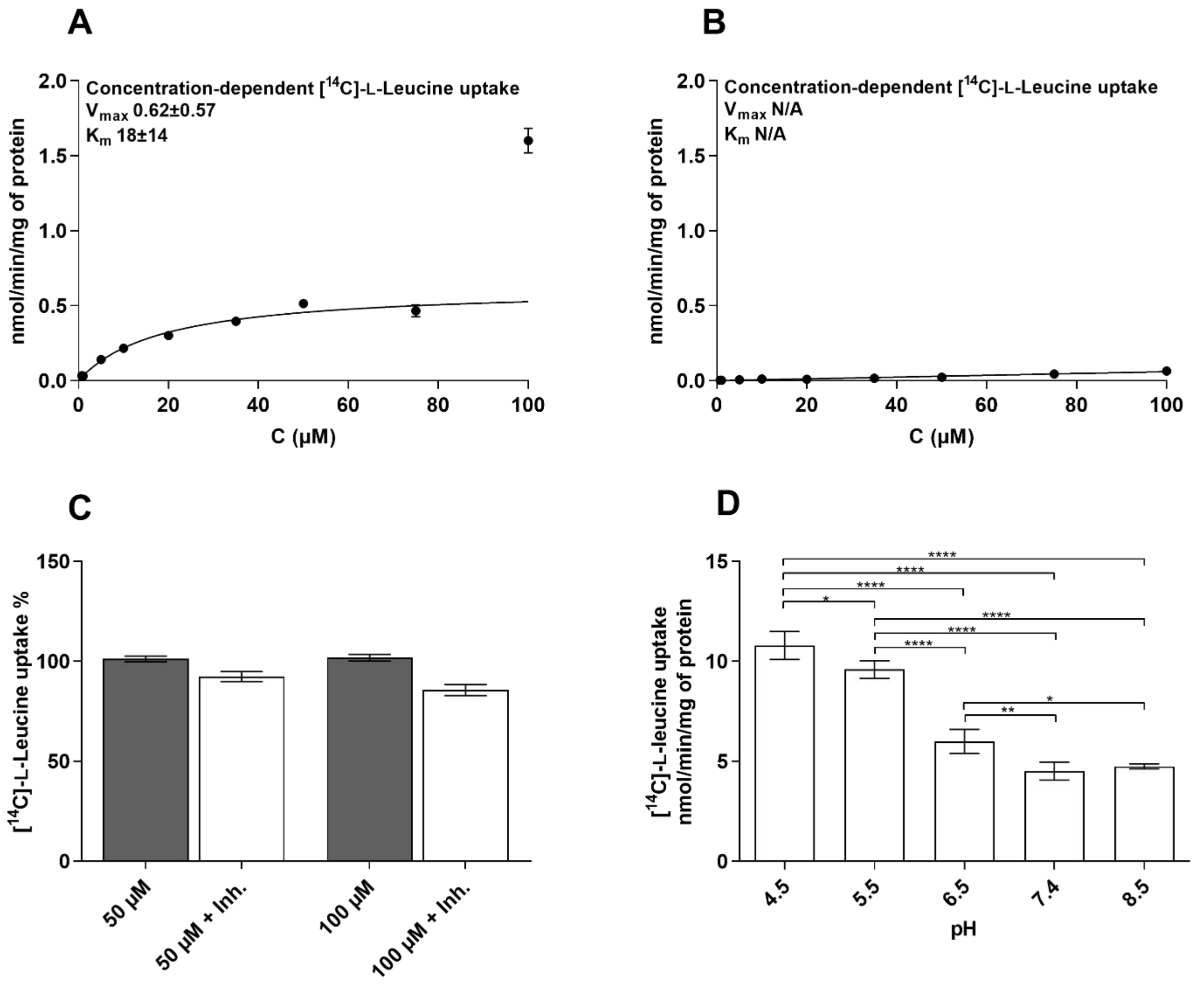

Figure S2. CAL27 transporter characterization. [^{14}C]-L-leucine uptake profiles: concentration-dependent (A), concentration-dependent in Na^+-free conditions (B), in the presence of a LAT1 specific inhibitor (C), and different pH conditions (D). The data is presented as mean ±SD, n=3 (**** P< 0.05, one-way ANOVA, followed by Tukey's multiple comparison test).

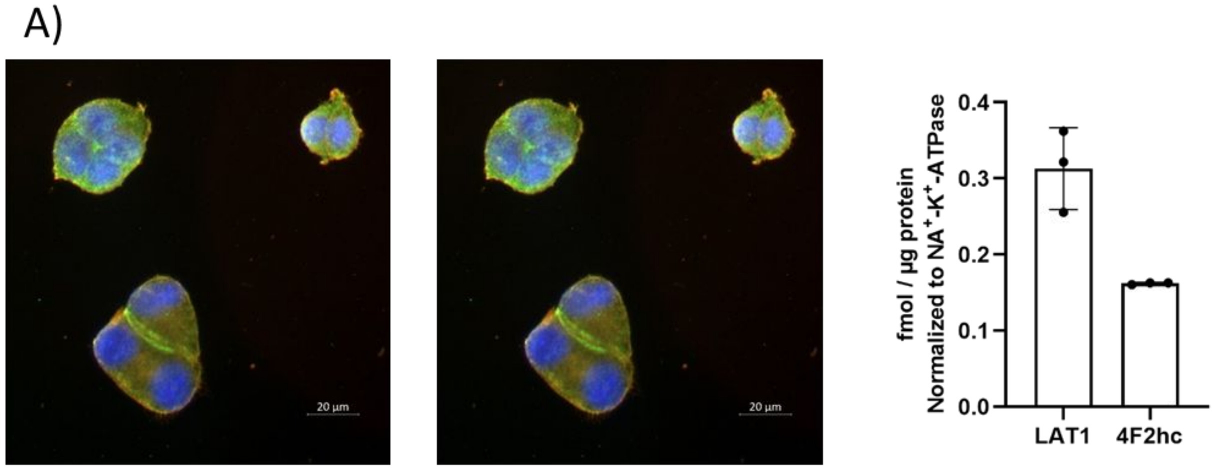

B)

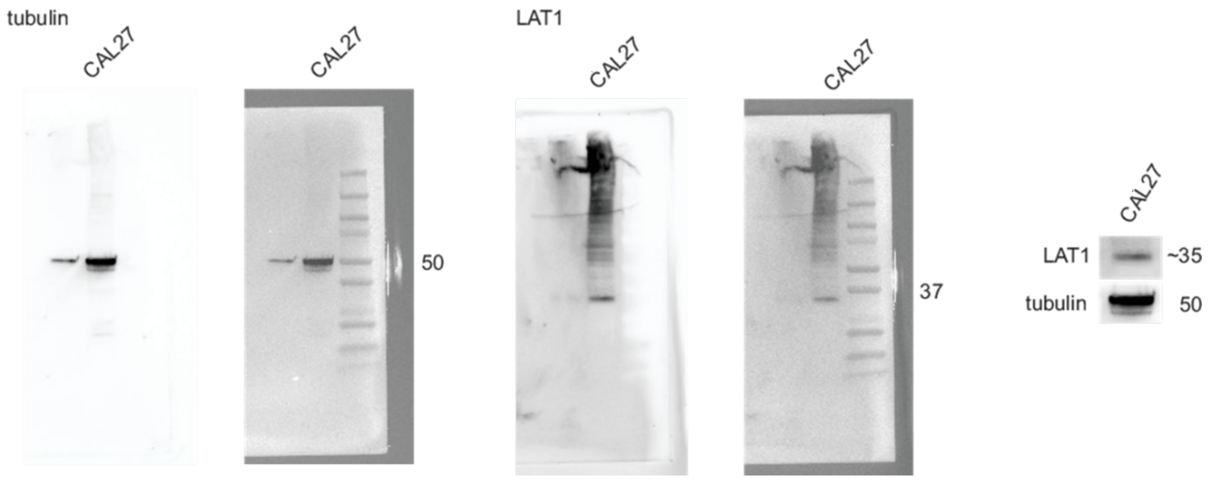

Figure S3. Fluorescence microscopy images after immunofluorescence staining, showing LAT1 localization in the cell membrane (LAT1= red, nuclei of the cells= blue, 4F2hc= green, overlapping of LAT1/4F2hc= yellow/orange) and the transporter protein amount expressed in CAL27 cells. (A) Western blot analysis of LAT1 protein expression in CAL27 cell line. (B)

3. Cytotoxicity study of the key compounds

Human colorectal adenocarcinoma Caco-2 cells were used to assess the cellular toxicity.[9] The cells were cultured in Dulbecco's modified Eagle medium containing 4.5 g/l glucose (DMEM, Gibco, Paisley, UK), supplemented with 10% fetal bovine serum (FBS , Gibco), 1% l-glutamine (Gibco), 1% non-essential amino acids (Bio-Whittaker, Belgium), 100 U/ml penicillin and 100 µg/ml streptomycin (Lonza Group Ltd., Basel, Switzerland). The cells (35 000 cells per well) were seeded in 48-well plates (Thermo Fischer Scientific, USA) 24 h prior to exposure and incubated at +37 °C (in 5% CO2).

Cell viability was assessed by MTT (3-(4,5-dimethylthiazole-2-yl)– 2,5-diphenyl-tetrazolium bromide) assay. Digitonin (0.2 mg/ml) was applied as a positive cytotoxicity control. After 24 h of seeding the cells, the culture medium was removed from the wells and replaced either with the studied compound or the control. The cells were exposed for 48 h in a cell culture incubator, with 5% CO_2 and a temperature of +37 °C.

After the chemical treatment, the incubation medium was removed, 150 µl of serum-free medium and 10 µl of 50 mg/ml MTT in 1xPBS were added. The plates were mixed gently and incubated at +37 °C (in 5% CO_2) for two hours. After the incubation, 100 µl if SDS-DMF-solution (pH 4.7) was added to solubilize the formazan crystals and the plates were further incubated at +37 °C (in 5% CO_2) overnight. The absorbances were measured at 570 nm with Multiplate Reader (Hidex, Finland).

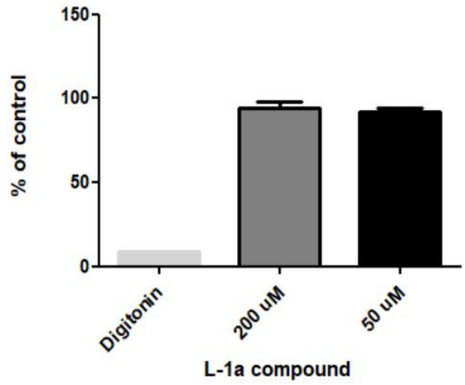

Figure S4. Exposure to L-5-boronotryptophan (**L-1a**) (at two concentrations 200 µM and 50 µM) did not had impact on cell viability in Caco-2 cells. Cell viability after 48-h exposure measured with MTT is shown as % of control ± SD, n = 4.

4. Supporting Figures from Molecular Dynamics Simulations

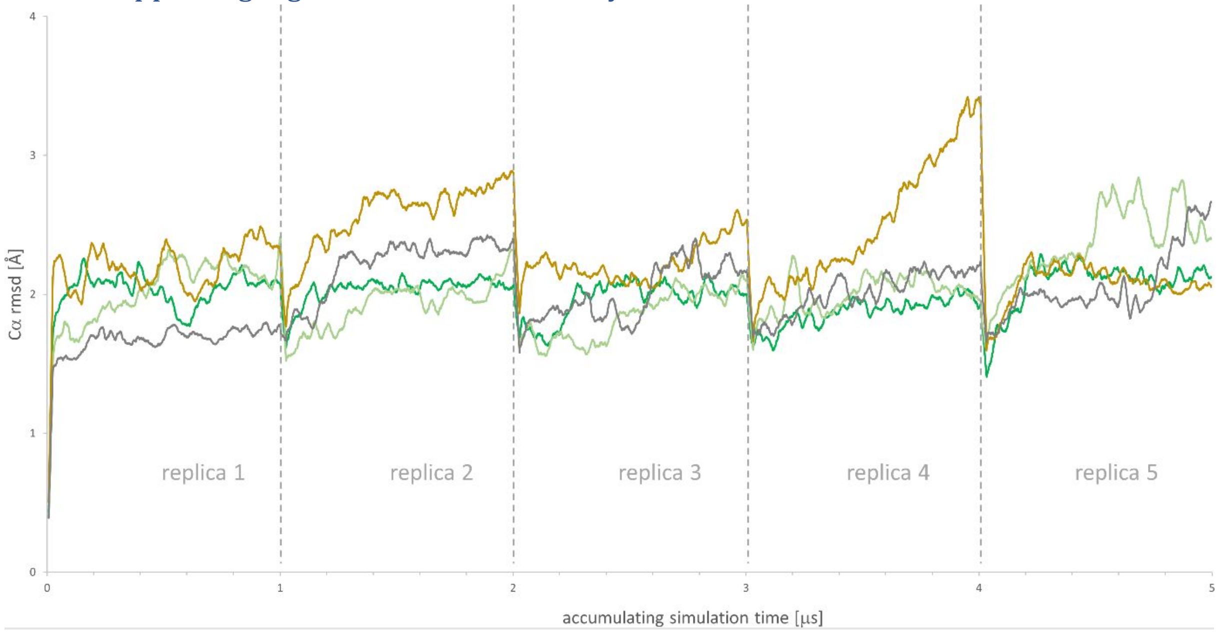

Figure S5. Root-mean square deviation (RMSD) of the studied LAT1 systems proving that simulations were stable along the replicated 5 μs simulations. Plot shows trendlines of the moving averages calculated over 25 structures (25 ns) for the RMSD data of Cα-atoms. Color coding is as follows: L-5-boronotryptophan in proximal pocket, light green; L-5-boronotryptophan in distal pocket, dark green; L-6-boronotryptophan, orange; and L-BPA, grey.

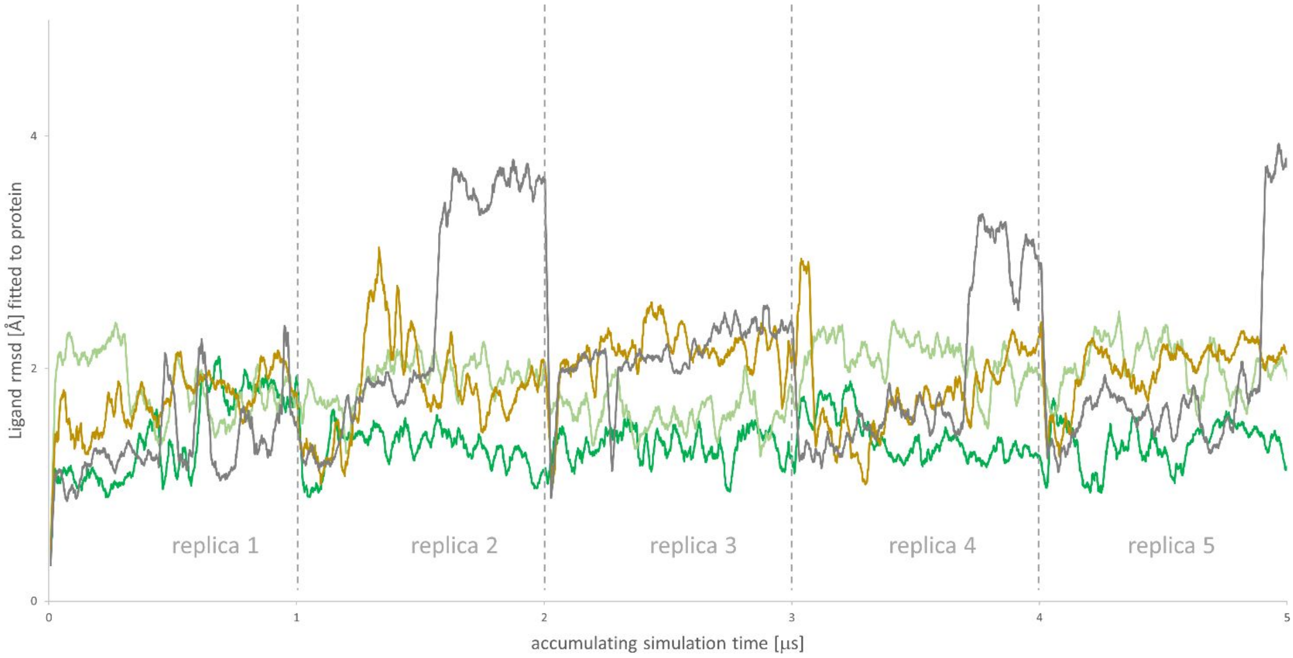

Figure S6. Ligand root-mean square deviation (RMSD) for each replicate is shown in Angstrom. The same color scheme as S4 has been used. L-5-boronotryptophan in proximal pocket, light green; L-5-boronotryptophan in distal pocket, dark green; L-6-boronotryptophan, orange; and L-BPA, grey. (Corresponding trendline settings used as in S4).

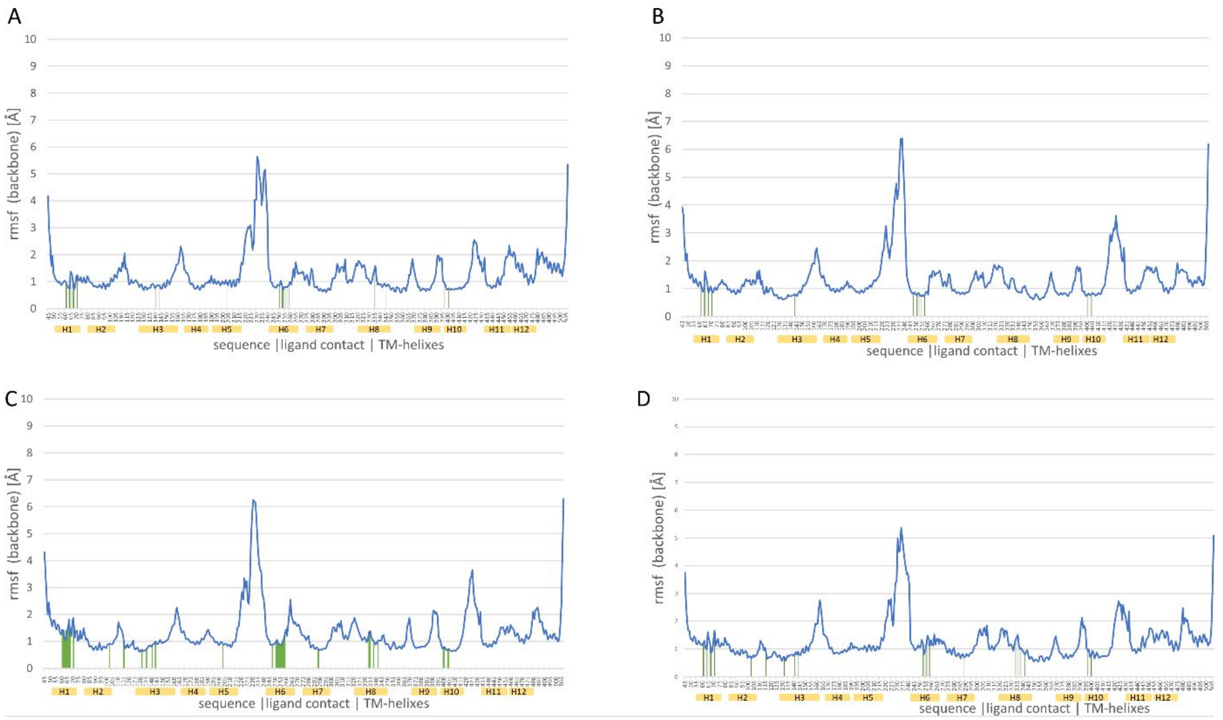

Figure S7. Residual protein backbone fluctuations (rmsf [Å]) and ligand contacts of L-5-boronotryptophan in proximal pocket (panel A), L-5-boronotryptophan in distal pocket (panel B), L-6-boronotryptophan (panel C), and L-BPA (panel D).

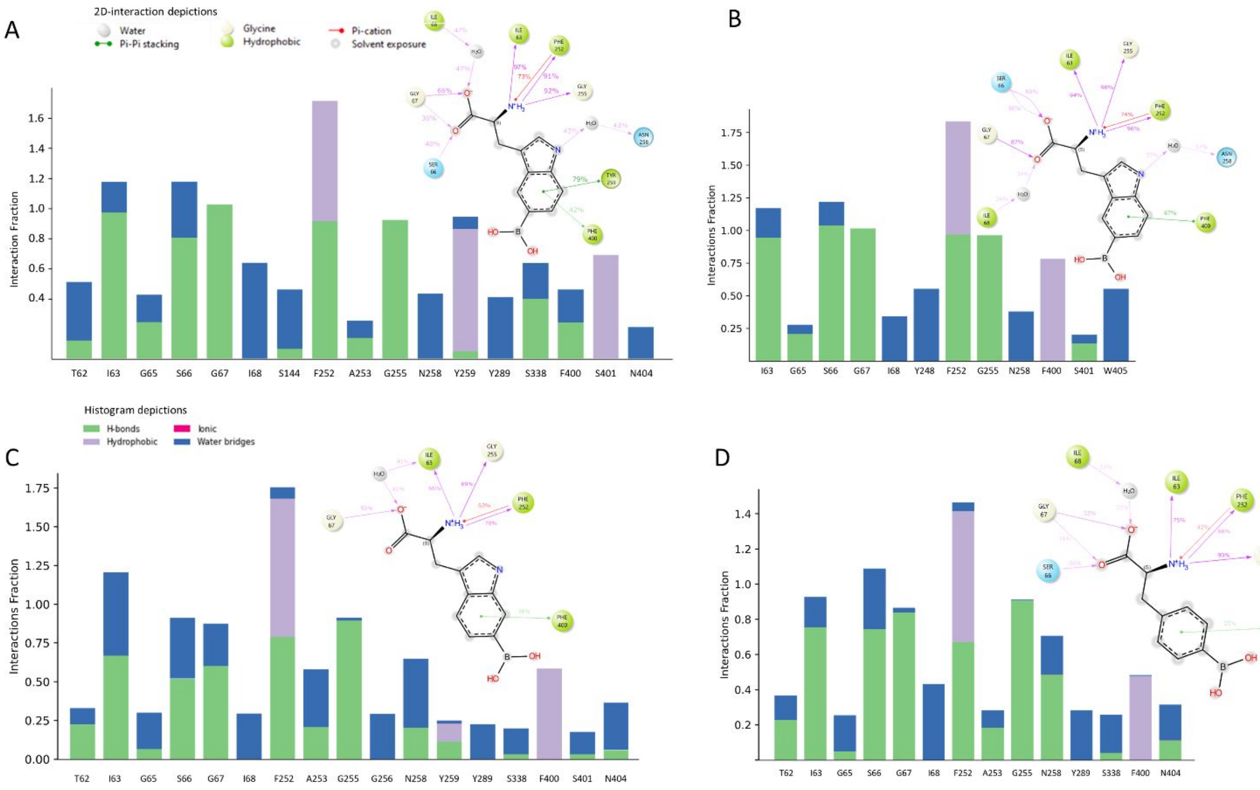

Figure S8. Most common Protein-ligand interactions (cut-offs used: 0.2 in histogram and 30% in 2D scheme) for L-5-boronotryptophan in proximal pocket (panel A), L-5-boronotryptophan in distal pocket (panel B), L-6-boronotryptophan (panel C), and L-BPA (panel D)

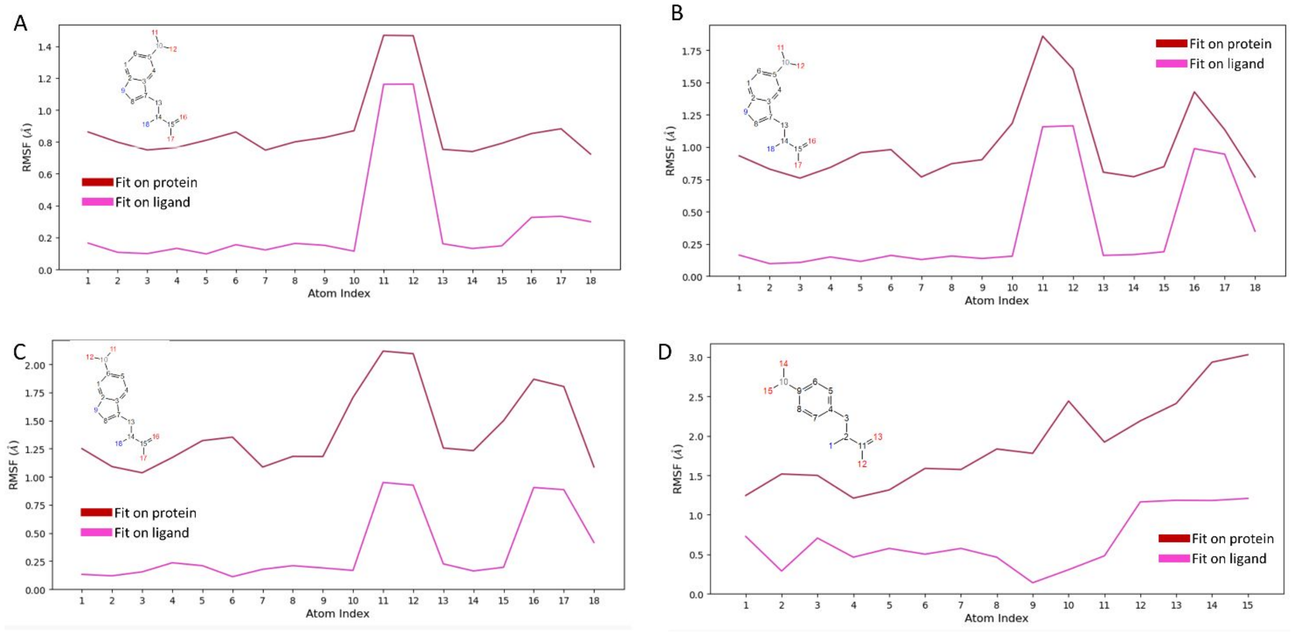

Figure S9. Ligand RMSF plot illustrating movable parts of studied ligands in the trajectories collected during replicated 5 μs of MD simulations. L-5-boronotryptophan in proximal pocket (panel A), L-5-boronotryptophan in distal pocket (panel B), L-6-boronotryptophan (panel C), L-BPA (panel D).

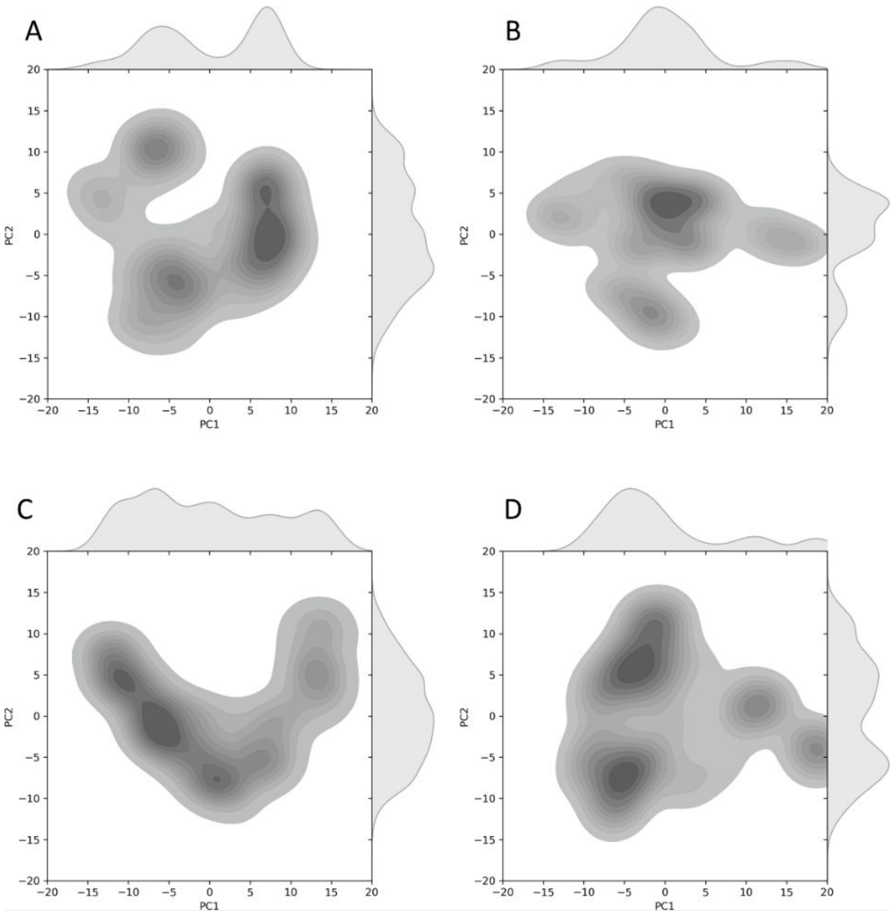

Figure S10. Principal component analysis (PCA) score plot of PC1 and PC2. Each system has been shown separately. L-5-boronotryptophan in proximal pocket (panel A), L-5-boronotryptophan in distal pocket (panel B), L-6-boronotryptophan (panel C), and L-BPA (D)

5. References

(1) Darses, S.; Michaud, G.; Genêt, J. P. Potassium Organotrifluoroborates: New Partners in Palladium-Catalysed Cross-Coupling Reactions. *European J Org Chem* **1999**, No. 8, 1875–1883. https://doi.org/10.1002/(sici)1099-0690(199908)1999:8<1875::aid-ejoc1875>3.0.co;2-w.

(2) Körner, C.; Starkov, P.; Sheppard, T. D. An Alternative Approach to Aldol Reactions: Gold-Catalyzed Formation of Boron Enolates from Alkynes. *J Am Chem Soc* **2010**, *132* (17), 5968–5969. https://doi.org/10.1021/ja102129c.

(3) Adamczyk-Woźniak, A.; Madura, I.; Velders, A. H.; Sporzyński, A. Diverse Reactivity of 2-Formylphenylboronic Acid with Secondary Amines: Synthesis of 3-Amino-Substituted Benzoxaboroles. *Tetrahedron Lett* **2010**, *51* (47), 6181–6185. https://doi.org/10.1016/j.tetlet.2010.09.091.

(4) Bartolucci, S.; Bartoccini, F.; Righi, M.; Piersanti, G. Direct, Regioselective, and Chemoselective Preparation of Novel Boronated Tryptophans by Friedel-Crafts Alkylation. *Org Lett* **2012**, *14* (2), 600–603. https://doi.org/10.1021/ol203216h.

(5) Murciano-Calles, J.; Romney, D. K.; Brinkmann-Chen, S.; Buller, A. R.; Arnold, F. H. A Panel of TrpB Biocatalysts Derived from Tryptophan Synthase through the Transfer of Mutations That Mimic Allosteric Activation. *Angewandte Chemie - International Edition* **2016**, *55* (38), 11577–11581. https://doi.org/10.1002/anie.201606242.

(6) Gynther, M.; Puris, E.; Peltokangas, S.; Auriola, S.; Kanninen, K. M.; Koistinaho, J.; Huttunen, K. M.; Ruponen, M.; Vellonen, K. S. Alzheimer's Disease Phenotype or Inflammatory Insult Does Not Alter Function of L-Type Amino Acid Transporter 1 in Mouse Blood-Brain Barrier and Primary Astrocytes. *Pharm Res* **2019**, *36* (1), 1–8. https://doi.org/10.1007/s11095-018-2546-7.

(7) Uchida, Y.; Tachikawa, M.; Obuchi, W.; Hoshi, Y.; Tomioka, Y.; Ohtsuki, S.; Terasaki, T. A Study Protocol for Quantitative Targeted Absolute Proteomics (QTAP) by LC-MS/MS: Application for Inter-Strain Differences in Protein Expression Levels of Transporters, Receptors, Claudin-5, and Marker Proteins at the Blood-Brain Barrier in DdY, FVB, And. *Fluids Barriers CNS* **2013**, *10* (1), 1–22. https://doi.org/10.1186/2045-8118-10-21.

(8) Pehkonen, H.; Filippou, A.; Väänänen, J.; Lindfors, I.; Vänttinen, M.; Ianevski, P.; Mäkelä, A.; Munne, P.; Klefström, J.; Toppila-Salmi, S.; Grénman, R.; Hagström, J.; Mäkitie, A. A.; Karhemo, P. R.; Monni, O. Liprin-A1 Contributes to Oncogenic MAPK Signaling by Counteracting ERK Activity. *Mol Oncol* **2024**, *18* (3), 662–676. https://doi.org/10.1002/1878-0261.13593.

(9) Pesonen, M.; Pasanen, M.; Loikkanen, J.; Naukkarinen, A.; Hemmilä, M.; Seulanto, H.; Kuitunen, T.; Vähäkangas, K. Chloropicrin Induces Endoplasmic Reticulum Stress in Human Retinal Pigment Epithelial Cells. *Toxicol Lett* **2012**, *211* (3), 239–245. https://doi.org/10.1016/j.toxlet.2012.04.002.
